# Biomimetic Sol–Gel Chemistry to Tailor Structure, Properties, and Functionality of Bionanocomposites by Biopolymers and Cells

**DOI:** 10.3390/ma17010224

**Published:** 2023-12-30

**Authors:** Yury Shchipunov

**Affiliations:** Institute of Chemistry, Far East Department, Russian Academy of Sciences, Vladivostok 690022, Russia; yas@ich.dvo.ru

**Keywords:** biomimetic, sol–gel, tetrakis(2-hydroxyethyl)silane (THEOS), tannic acid, immobilization, bionanocomposite, hybrid, biocatalyst, biosensor, photonic

## Abstract

Biosilica, synthesized annually only by diatoms, is almost 1000 times more abundant than industrial silica. Biosilicification occurs at a high rate, although the concentration of silicic acid in natural waters is ~100 μM. It occurs in neutral aqueous solutions, at ambient temperature, and under the control of proteins that determine the formation of hierarchically organized structures. Using diatoms as an example, the fundamental differences between biosilicification and traditional sol–gel technology, which is performed with the addition of acid/alkali, organic solvents and heating, have been identified. The conditions are harsh for the biomaterial, as they cause protein denaturation and cell death. Numerous attempts are being made to bring sol–gel technology closer to biomineralization processes. Biomimetic synthesis must be conducted at physiological pH, room temperature, and without the addition of organic solvents. To date, significant progress has been made in approaching these requirements. The review presents a critical analysis of the approaches proposed to date for the silicification of biomacromolecules and cells, the formation of bionanocomposites with controlled structure, porosity, and functionality determined by the biomaterial. They demonstrated the broad capabilities and prospects of biomimetic methods for creating optical and photonic materials, adsorbents, catalysts and biocatalysts, sensors and biosensors, and biomaterials for biomedicine.

## 1. Introduction

Silicon is the second most abundant element in the Earth’s crust [1,2]. It is the most important inorganic component of natural waters, used as a building material and as food by living organisms. Its greatest quantities are accumulated by diatoms, which constitute one of the largest groups of phytoplanktons. They annually assimilate ~7 trillion kg of silicon in the form of orthosilicic acid, synthesizing frustule from amorphous silica, which is called biogenic silica or biosilica [3,4]. The formation of biosilica occurs under normal conditions, neutral pH, and under superior control of biopolymers, which completely regulate biosilicification processes and its highly ordered hierarchical structural organization with an impressive geometry [5]. The total industrial production of silica materials is at the level of 10^9^ kg [6], i.e., the amount produced is three orders of magnitude less than biosilica in living nature. More important is the fact that anthropogenic and geological syntheses, unlike biosilicification, take place at high temperatures, pressures, and inconvenient pH. Under such conditions, biopolymers and living cells cannot be involved in the regulation of these processes.

Biopolymers created by nature over a long evolution—proteins, enzymes, polysaccharides, DNA, polyphenols and antibodies/antigens—are of great interest for the obtaining of materials for biomedicine, medical diagnostics, biocatalysis, biosensorics, proteomics, genetic engineering, biotechnology, bioremediation, food, cosmetics and energy due to their unique functional properties, functioning at ambient temperatures and in aqueous environments with high efficiency. Successful use of a biomaterial is possible after entrapment in biocompatible matrices, in which intactness and functional properties are fully preserved [7,8,9,10,11,12,13,14,15,16,17,18]. One of the widely used approaches is sol–gel technology [19,20,21,22,23,24,25,26,27,28,29,30,31]. During the synthesis, biopolymers are mineralized and hybrid structures with a nanosized inorganic component are formed [10,18,32,33,34]. It is suggested to categorize such hybrid materials as bionanocomposites [35].

Sol–gel technology has a number of undoubted advantages, which made it popular for the silicification of biomaterial [9,20,22,26,30,31,36,37,38,39,40,41,42,43,44]. These include control over synthesis conditions—pH, salt composition, additives of various substances, and temperature. The processes take place in an aqueous environment. They do not consume much energy and do not require expensive and complex equipment. The shape, morphology, porosity, charge, and composition of the silica are controlled and can easily be tailored to specific requirements. The formed silica matrix, as in the case of diatoms, increases the tolerance of the entrapped biomaterial to harsh external conditions and chemicals and protects against biodegradation caused by microorganisms. Synthetic amorphous silica and silicic acid is a non-toxic material [45] classified by the United States Food and Drug Administration as “Generally Recognized as Safe”, the use of which is approved as a food additive by the European Food Safety Authority [12,17,46,47].

The use of conventional sol–gel chemistry for entrapping biopolymers and living cells into the silica matrix encounters a whole range of problems. They are caused both by the conditions of the procedure and by the poor compatibility of precursors with the biomaterial.

Well-established sol–gel technology is based mainly on the commercially available precursor tetraethoxysilane, also known under the abbreviation TEOS [37,38,48]. Its use for the immobilization of biopolymers faces such major disadvantages as the release of ethyl alcohol during hydrolysis, as well as the addition of it or another organic solvent to dissolve hydrophobic TEOS, carrying out sol–gel synthesis in acidic or alkaline media and using heating to accelerate the processes. The noted factors have an unfavorable effect on the intactness and functional properties of biopolymers, as well as cells [7,20,22,49,50,51,52,53]. In particular, proteins denature, enzymes, accordingly, lose activity, and many polysaccharides precipitate, which leads to the formation of amorphous mass that does not have a certain structural organization and does not possess the functions of the entrapped biomaterial.

The fundamental difference between the results of mineralization in vitro and biomineralization in diatoms forced researchers to look for approaches to improve the compatibility of the precursor and the conditions for performing sol–gel synthesis for the immobilization of biomaterial. Processes in living cells are considered as a model to follow, which implies a significant change in the synthesis protocols in the laboratory, making them similar, biomimetic to biosilicification.

The sol–gel method is classified as biomimetic if the process is also fully compatible with biopolymers; it is controlled and manipulated in full measure by them both at the stage of reactions and the formation of hybrid structures with a certain structural organization. The impetus for the development of such approaches came from studies at the end of the last—beginning of this century [54,55,56,57,58,59], in which proteins responsible for biosilicification in diatoms and glass sponges were identified and then isolated. The possibility of obtaining bionanocomposites with a controlled structure under the conditions under which biosilica is synthesized was demonstrated. Further studies included both the compatible precursors matching and the elimination of the use of acids/alkalis, organic solvents, and heating.

The objective of this review is to critically analyze the proposed biomimetic approaches for biopolymer and cell encapsulation, as well as the biopolymer-tailored structure and properties of synthesized bionanocomposites. First, the basics of traditional sol–gel chemistry and its limitations that hinder biomimetic applications are briefly reviewed. The main features and proteins responsible for biosilicification in diatoms and glass sponges are noted, as well as in vitro experiments with them, which confirmed their unique role and indicated the possibility of creating biomimetic approaches. The main pioneering studies in this area, which laid its foundations and then successfully developed, are noted. The advantages of sol–gel technology are listed, which are important for the fabrication of new hierarchically structured functional nanomaterials. The advantages and disadvantages of currently known precursors and protocols proposed for the immobilization of biopolymers and cells are critically analyzed. In conclusion, some areas of application of biomimetically synthesized bionanocomposites are considered. To our knowledge, such a comprehensive and thorough review of the most important aspects has not yet been conducted. Consideration was usually limited to specific issues.

## 2. Chemistry of Sol–Gel Processes

The transformation of a solution into a gel-like state as a result of the combination of sol nanoparticles into a three-dimensional network due to physical interactions or the formation of covalent bonds is called sol–gel transition or sol–gel processing [36,37,38,48,60,61,62]. It lies at the basis of many technological processes known as the sol–gel technology. The sol–gel transition is also widespread in the surrounding world and in living nature. Thanks to its simplicity, low temperatures, the use of aqueous solutions and simple equipment, sol–gel technology is used in industry for the production of adsorbents, catalysts, ceramics, glasses, membranes, fibers, fine powders, microparticles, nanocomposites, insulating materials, protective coatings, and so on.

### 2.1. Silicic Acid

Sol–gel synthesis of silica is based on the strong tendency of orthosilicic or silicic acid to the condensation reaction [2,36,37,38,63,64]:

(OH)_3_Si-OH + HO-Si(OH)_3_ → (OH)_3_Si-O-Si(OH)_3_ + H_2_O, (1)

as a result of which siloxane bond Si-O-Si is formed. The two silicon atoms are bonded covalently to each other via oxygen. The remaining three sylanol groups Si-OH at each silicon atom are involved in the following condensation reactions:

(OH)_3_Si-OH + (OH)_3_Si-O-Si(OH)_3_ + HO-Si(OH)_3_ → (OH)_3_Si-O-Si(OH)_2_-O-Si(OH)_2_-O-Si(OH)_3_ + H_2_O, (2)

which lead to polymerization and the formation of polysilicic acids. At the initial stage, oligomeric reaction products are formed in the form of sol particles. Their flocculation, which promotes the formation of covalent siloxane bonds between them, leads to a sol–gel transition with the formation of a three-dimensional network structure.

The tendency of silicic acid to be polymerized, i.e., its instability, does not allow it to be stored for a long time and used as needed [36,37,38,52]. It must be used within 24 h after preparation. Therefore, silicic acid is prepared immediately before obtaining silica. Substances that are taken for its synthesis are called precursors. These include sodium metasilicate and alkoxides. They are discussed in the next sections.

### 2.2. Sodium Metasilicate

Van Helmont in 1640, by calcining silica minerals with sodium and potassium carbonates, obtained a new compound called “water glass” (cited from [30]), which began to be produced in Europe and America by 1855 [36]. The water-soluble form is currently available as sodium metasilicate Na_2_SiO_3_. Because it is a salt of a weak acid and a strong base, aqueous solutions are alkaline (pH 11–13). They are stable and keep well. Acidification leads to the release of silicic acid, which enters into polycondensation reactions (2) [2,36]. The formed polysilicic acids cause gelation of solutions. The chemical reaction in general can be presented as follows:

Na_2_SiO_3_ + 2HCl + (*x* − 1) H_2_O → SiO_2_ × *x*H_2_O + 2NaCl. (3)

The advantages of Na_2_SiO_3_ are availability, since it is produced industrially, low cost and ease of silica synthesis. The main disadvantage is significant amounts of sodium salt, which, as can be seen from the above reaction equation, is found in the reaction products. Its presence can create certain problems in some cases. In such a situation, the sodium salt is removed by dialysis. For this purpose, an ion exchange resin can be used [21,65,66], for example, Dowex. When sodium cations are exchanged for a proton, acidification occurs, which leads to polycondensation of the resulting silicic acid and the formation of a sol. Additional procedures lengthen and complicate the production of silica. It is also believed that in the case of sodium metasilicate there are very limited possibilities for controlling the structure of the synthesized silica [26,27,67].

### 2.3. Alkoxides

**Figure 1:**
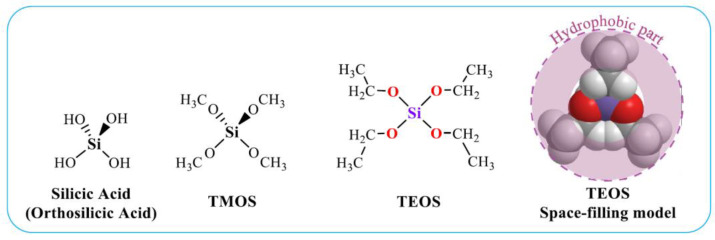
Structural formulas of silicic acid and two main precursors used in sol–gel technologies. TEOS is also presented as a space-filling model. The hydrophobic part of the molecule is highlighted.

Alkoxides Si(OR)_4_ are silicon ethers. The most famous of them are tetraethyl orthosilicate or tetraethoxysilane (TEOS) and tetramethoxysilane (TMOS), containing, respectively, ethanol and methyl alcohol residues [37,38,62,68,69,70,71]. Their structural formulas are shown in Figure 1. TEOS and TMOS provide greater opportunities for the formation of silica materials than sodium metasilicate [26,38,52,67]. When using them, there is no release of sodium cations. Alkoxides allow the formation of silica with different porosities and morphologies, which are regulated by the precursor and synthesis conditions. Therefore, they are more widely used than Na_2_SiO_3_ for the formation of bionanocomposites. If TMOS and TEOS are compared, the former hydrolyzes faster than the latter. However, the release of methanol poses more problems due to its greater toxicity in comparison with ethanol. It has to be deleted [72].

Precursors, upon contact with water or vapor absorption, as first established by Ebelmen [73], hydrolyze in accordance with the reaction equation:

Si(-O-CH_2_-CH_3_)_4_ + n H_2_O → (HO-)_n_Si (-O-CH_2_-CH_3_)_4−n_ + n HO-CH_2_-CH_3_, (4)

In the limiting case, all four ethanol residues are separated, which leads to the formation of silicic acid Si(OH)_4_, of which TEOS is a derivative. Its formation can only occur in very dilute solutions. At the concentrations at which sol–gel syntheses are carried out, silicic acids enter into condensation reactions to form oligomeric polysilicic acids.

It is more likely that the process does not reach the silicic acid formation. Most probably silanol group(s) Si-OH, formed as a result of nucleophilic substitution of alkoxy group (-OR) for a hydroxyl one (-OH), are involved in condensation reactions due to their high reactivity. In this case, two types of reactions are possible, occurring according to the oxolation or olation mechanism. The former occurs through condensation of two sylonol groups as a result of the exchange of hydroxo ligand for “oxo” one [37,38,62]:

(RO-)_4−n_ Si (-OH)_n_ + (HO-)_n_Si (-OR)_4−n_ ⮀ (OH)_n−1_(RO-)_4−n_ Si-O-Si (-OR)_4−n_ (OH)_n−1_ + H_2_O. (5)

The olation mechanism occurs as a result of the interaction between sylanol and alkoxy groups in accordance with the following general reaction:

Si (-OR)_4_ + (HO-)_n_Si (-OR)_4−n_ ⮀ (RO-)_3_Si-O-Si (-OR)_4−n_ (OH)_n−1_ + HO-R, (6)

In both cases, a dimer is formed in which two silicon atoms are bonded to each other by siloxane bond Si-O-Si.

Replacement of the remaining alkoxy groups in the molecule with hydroxyls leads to the involvement of the dimer in condensation reactions (5) or (6), as a result of which polysilicic acid is formed. The final product is partially hydrated silica SiO_2_ × *x*H_2_O, known as silica. It consists of a randomly arranged three-dimensional network of tetrahedral silicon atoms Si(O-)_4_ connected by siloxane bonds. The number of the latter varies from 1 to 4. Therefore, the silica structures always contain silanol groups Si-OH, which can also be found in ionic form Si-O^−^ H^+^ or Si-O^−^ Na^+^, if sodium metasilicate was taken in the synthesis.

### 2.4. Disadvantages of the Traditional Sol–Gel Synthesis

**Figure 2:**
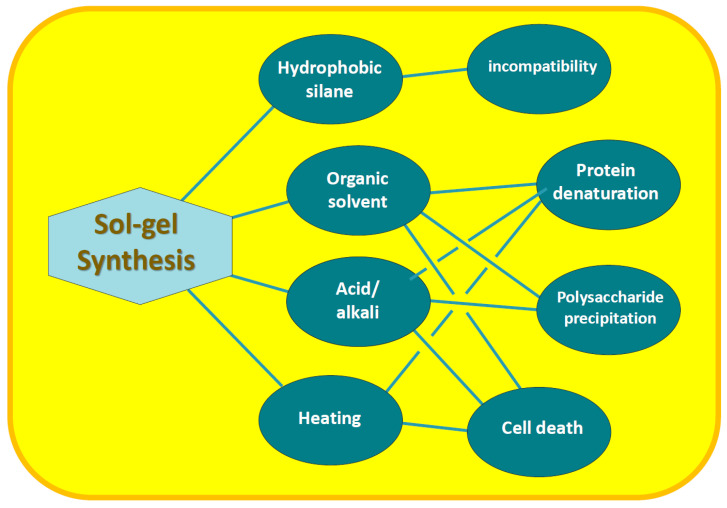
Disadvantages of sol–gel synthesis and their consequences for biomaterial immobilization.

Alkoxides used for silicification of biopolymers and living cells, as well as the conditions of the sol–gel process, are unfavorable for the inclusion of most of the biomaterial, which limits their widespread use for its immobilization [7,9,20,22,49,74,75,76,77,78,79,80]. The disadvantages include the following, presented schematically in Figure 2 as well.

1. Hydrophobicity of alkoxides. The TMOS and TEOS molecules, as seen in Figure 1, are surrounded by the methyl groups of methanol and ethanol residues, respectively, with hydrocarbon chains oriented outward. Their presence on the surface makes it nonpolar, which makes the precursor hydrophobic. Its consequence is poor solubility in aqueous solutions and lack of contact with biomacromolecules and cells, which is needed for their mineralization [53,81,82,83];
2. Organic solvent. Hydrolysis of TMOS and TEOS is accompanied by the release of alcohol (reaction (4)), the content of which can reach 35% *v*/*v* [51]. In addition, an organic solvent—usually alcohol—is introduced to achieve complete dissolution of the silanes in the reaction mixture. Methyl or ethyl alcohols are most often added. They denature proteins, deactivate enzymes [84,85,86,87], precipitate polysaccharides and cause cell membrane lysis [5,10,88]. Negative effects on RNA aptamers were also noted [66];
3. Acid and alkali. Acidification or alkalization of the reaction mixture is explained by the specificity of the reactions of hydrolysis (4) and condensation (5) and (6). The first proceeds faster in the acidic region, and the second—in the neutral and alkaline (section). If the synthesis is carried out at neutral pH values, optimal for the biomaterial, then the formation of silica will last at least for weeks [62]. The process is sharply accelerated by the addition of acid or alkali, which are considered catalytic additives [36,37,38,62,69]. Acidification/alkalinization leads to denaturation of proteins, precipitation of carboxyl-containing polysaccharides and chitosan [89,90,91];
4. Heating. Condensation reactions (5) and (6) are reversible, which slows down the formation of silica. To shift the equilibrium towards its formation and hasten the process, the reaction mixtures are heated [36,37,38,62,69]. Heating is accompanied by denaturation of proteins, loss of enzyme activity and death of cell cultures [84,92];
5. Syneresis. Condensation reactions after gelation, although they slow down sharply, still continue. This leads to further cross-linking and compaction (syneresis) of the silica matrix with time [36,37,38,62,69]. Shrinkage can reach 85% [51,75,93]. Syneresis has a negative effect on immobilized proteins and cells. In particular, it leads to loss of global and segmental motion by proteins as aging proceeded [51]. A gradual decrease in the binding of human serum albumin ligands was noted, which after 2 months amounted to only 15% of the initial value exhibited by the protein in solution [94]. Shrinkage and damaging cells are noted [5], limiting proliferation until it stops [44].

The noted negative impact of TMOS/TEOS and the conditions of well-established sol–gel synthesis on biomaterial does not allow it to be extended to the mineralization of most biopolymers and microorganisms, as well as to the formation of bionanocomposites. There are only isolated examples of its successful application. One exception is the immobilization of lipases, which can function in non-aqueous media, including alkanes (see Section 3).

## 3. Biomineralization

Among the chemical elements, silicon is the second most abundant in the Earth’s crust [1,2]. It is also widely distributed in living matter in the form of a silica, called biogenic silica or biosilica [95]. Davy, in a monograph published in 1815, drew attention to its presence in plants. He found that the SiO_2_ content in the leaf ash of some samples reached 70% [96]. Further studies confirmed the fact of silica accumulation by plants, among which rice, grains and cucumbers stand out [97,98]. Its main function is to mechanically strengthen aerial parts, but SiO_2_ appears to be more vitally important, participating in growth and defense processes, for example, cadmium detoxification in rice and the alleviation of abiotic stress damage [97,98,99,100,101,102,103,104,105]. In addition to plants, silica is also found in animals and microorganisms, acting as a nutrient that is important for their growth and functioning [3,4,106,107,108,109,110].

**Figure 3:**
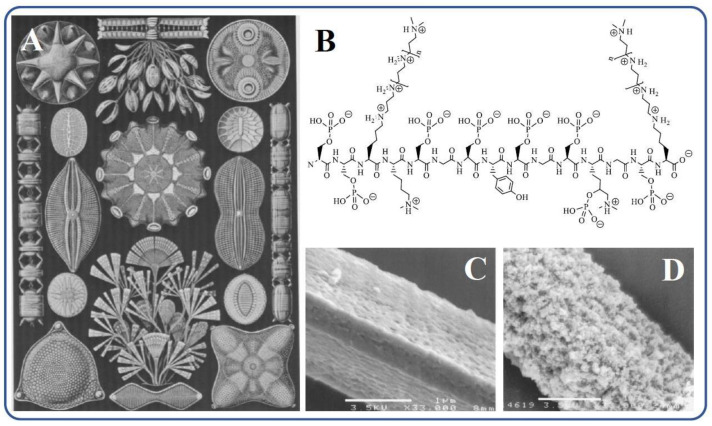
(**A**) Drawings of diatoms [116]. (**B**) Schematic chemical structure of silaffin-1A_1_ [115,117]. (**C**) Silicatein filament prepared for silicification and (**D**) after the silicification in vitro in [56].

The largest amounts of biosilica are synthesized by marine organisms: diatoms, sponges. radiolarians, and silicoflagellates [3,4,106,107,108,109]. They absorb silicic acid from water, which is transformed into silica. The process is called biosilicification, which is a special case of biomineralization. Diatoms—microscopic, single-celled algae—are considered the dominant producers of biosilica [4,106,111,112]. There are ~20,000 species, the size of which varies from 2 to 200 μm (Figure 3A) [113]. They annually produce ~7 trillion kg of SiO_2_, which forms rigid cell walls (frustules) [3,4,114]. The study of *Cylindrotheca fusiformis*, which serves as the most extensively studied “model” diatom, isolated its protein components. Among them were polycationic proteins called silaffins. They mediated biosilicification processes, and when added to aqueous solutions of silicic acid, they sharply accelerated sol–gel processes, causing the precipitation of polysilicic acids within seconds [54,57,59,115]. Silaffins contained mainly serine and lysine residues. As can be seen from the structural formula in Figure 3B, oligo-propyleneimine chains are attached to the ε-amino groups of the latter.

In parallel with diatoms, independent studies of marine sponges were carried out. The biogenic silica in them is located in needlelike spicules, serving to support the body and protect against predation. In the central part of the silica spicules, there is a channel with a diameter of up to 1–2 μm, filled with proteinaceous axial filament (Figure 3C), which consists of 70% protein, called silicatein [55]. Biosilica is located around in the form of concentric layers 0.3–2 μm thick [109,118,119,120,121]. In a model experiment in vitro in which isolated silicatein was introduced into a TEOS solution with pH 6.8, the formation of SiO_2_ was observed, which confirmed its participation in biosilicification in marine sponges (Figure 3D). Albumin, trypsin, papain, or BSA under the same conditions almost did not accelerate the sol–gel process, which proceeded very slowly [56]. In addition, SiO_2_ deposition was not observed on cellulose fibers that replaced silicatein filaments [56]. Additional confirmation of the catalysis of silicatein hydrolysis and condensation reactions, which could not be studied separately, is also given in [122]. Povarova et al., who conducted experiments with tetra(glycerol)orthosilicate, showed that there was at least a 20-fold acceleration of sol–gel processes. In this case, the silane condensed on the protein, apparently in the region of the active center, undergoing further transformations.

The identification and isolation of proteins responsible for the synthesis of biosilica in diatoms and glass sponges have allowed significant advances in the understanding of biosilicification processes. The amino acids contained in their composition were identified. They served for the synthesis of polypeptides that could replace the hard-to-separate silaffin and silicatein and perform a similar catalytic role in the synthesis of SiO_2_. Among the first works conducted in this direction, the following should be noted:

- Cha et al. [123] initially studied polypeptide cysteine, since the amino acid is part of silicatein, but the catalytic effect was not observed. They explained its absence by the poor solubility of the polypeptide in water. The synthesized block copolypeptides with a water-soluble polylysine block turned out to be catalytically active. Copolypeptides self-assembled into aggregates which determined the hydrolysis of TEOS and the formation of SiO_2_ of different morphology;
- Patwardhan et al., in a series of papers [124,125,126,127,128,129,130], showed that cationic polymers and peptides, including poly-lysine, -arginine, -histidine, -allylamine, and -amine, catalyzed the sol–gel transition in solution with preformed SiO_2_ sol, acting as the template They obtained spherical particles having fiber-like and ladder-shaped silica morphologies with periodic voids;
- Livage and coworkers independently obtained similar results with polylysine and polyarginine [131,132], as well as with an arginine-containing surfactant [133]. They observed the gelation of solutions containing silica oligomers and the precipitation of silica sols;
- Naik et al. [134] examined the silicification provided by polypeptides, using amino acids derived from silaffin. They formed silica with different morphologies, which ranged from spherical to fibrillar.

It should be noted that studies by Patwardhan et al., Livage et al., and Naik et al., as well as other authors [5,76,135,136,137,138,139,140,141,142,143,144,145,146,147,148], were carried out in neutral solutions, but they took pre-prepared silica sols obtained by hydrolysis of TEOS or TMOS. When TEOS was used in the experiment without preliminary hydrolysis, 90% of the calculated amount of SiO_2_ in the presence of polylysine at pH 6.9 was formed only after ~20 days [149]. In the case of polyserine and polycysteine, the processes proceeded even more slowly. In particular, polycysteine encouraged the formation of only 10% silica over the same period of time. The results correlate with the conclusions of [150], in which the formation of silica at pH 11.6 was observed after 2 weeks.

Acceleration of sol–gel processes at neutral pH values, including reactions of hydrolysis and condensation of alkoxides, was observed only in one case, when the silicatein protein was taken [56]. Similar catalytic activity in vitro was not observed for silaffin [54,57,59,115]. In studies on the immobilization of various enzymes, it was discovered that lipases, which do not participate in biosilicification processes in living cells, are capable of accelerating sol–gel processes at neutral pHs [151,152,153,154,155,156]. However, the first attempts to incorporate the enzyme into a silica matrix using the conventional method using TMOS were not successful [151,157]. The immobilized lipase had low enzymatic activity. The situation changed drastically when TMOS was taken together with methyltrimethoxysilane CH_3_Si(OCH_3_)_3_. Immobilization carried out by a combination of two precursors led to a 13-fold increase in lipase activity compared to the initial level in solution [151]. A possible explanation is that lipases, upon reaching the phase boundary, enter an active state as a result of a conformational rearrangement [158,159]. The phenomenon is called “interfacial activation”. This evidently occurs upon immobilization in a silica matrix with a hydrophobic surface coated with methyl groups [160,161]. Active lipases catalyze the hydrolysis of esters. They exhibit hydrolytic activity, as shown in [156], and towards alkoxides, catalyzing sol–gel processes not only at the stage of hydrolysis but also condensation [26,155,156,162,163]. The similarity in the action of lipases and silicatein may be explained by the composition of the active site. They both contain the amino acid serine in significant quantities [158,159].

**Figure 4:**
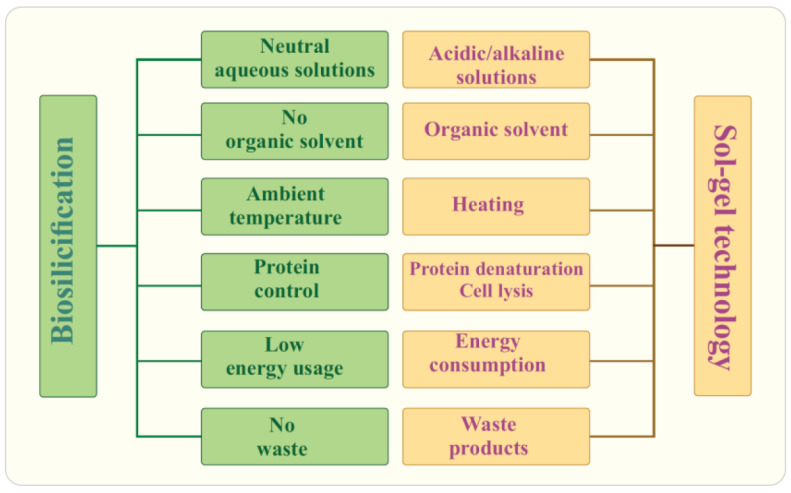
Biosilicification versus sol–gel technology.

Research on biosilicification in the living matter has revealed fundamental differences from well-established sol–gel technology. They are summarized in Figure 4. Biosilica in diatoms and marine sponges is formed under mild conditions and green routes. Its synthesis occurs under complete control with the participation of proteins and is genetically predetermined [4,6,97,98,114,164,165,166,167,168]. Shapes, sizes, structure, and meso/microporosity are reproduced from generation to generation, have an intricately and ornately ordered hierarchical structural organization, and, in some cases, such as diatoms, an impressive, sophisticated architecture (Figure 3A). It is important to note that the formation of biosilica occurs at a high rate, although the concentration of silicic acid in natural waters is insignificant. In seawater, it is at a level of 10 μM, in freshwater—100 μM [36,111,113]. This content is significantly lower than the critical value (~0.002 M [169]), from which the polymerization of silicic acid begins. Moreover, in diatoms, which have the ability to concentrate SiO_2_ from dilute solutions at a high rate [111,170], it proceeds ~100 times faster than abiotic—geochemical and industrial—syntheses [171]. This means that biosilicification is a highly efficient process that also does not require much energy and does not cause environmental pollution. The forming diatom cell wall is an almost pure silica (~97% [138]), which has high mechanical strength, providing the cells with the necessary protection [14,112].

## 4. Historical Background of Biomimetic Synthesis

Sol–gel science has a long history dating back hundreds of years. The starting point is often taken to be 1864, when an article by Graham appeared, describing experiments with silica sols and coining the term “sol–gel” [172]. Van Helmont, 200 years before this date—in 1640—discovered “water glass” by calcining silica minerals with potassium or sodium carbonates, and then found that upon acidification precipitation occurred, i.e., observed a sol–gel transition (citation according to [30]). Ebelmen in 1846 synthesized TEOS, during the study of which he described the main features of sol–gel chemistry with alkoxide. In particular, when introduced into water, precipitation of the formed silica was observed, and when the silane came into contact with air, vapors were absorbed, which led to the formation of a transparent glassy mass [73].

**Figure 5:**
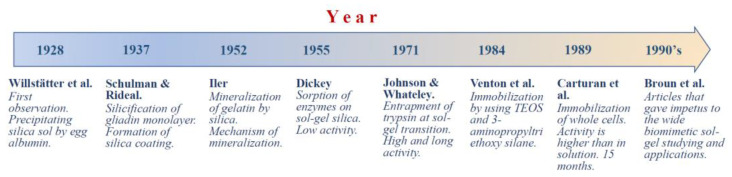
Chronology of the first works related to biomimetic mineralization of biomaterial [173,174,175,176,177,178,180,182].

The history of biomimetic sol–gel chemistry is not so long. Pioneer works in chronological order are shown in Figure 5. One of the first to become interested in the effect of biopolymers on a silica sol solution was Willstätter, Kraut, and Lobinger [173]. They added egg albumin, which resulted in coprecipitation. The phenomenon was not studied, but only the fact of the interaction of the protein with the silica sol was established.

Silicification of biopolymers in vitro was clearly established by Schulman and Rideal in 1937 in experiments with monolayers of gliadin, which is one of the two main components of the gluten [174]. They showed that silicic acid introduced into the solution reacted with the protein monolayer, eventually forming an impermeable film on its surface. Later, Clark et al., who studied monolayers of albumin and insulin, confirmed the interaction of silicic acid with proteins [169]. They also noted that Si(OH)_4_ adsorbed onto protein monolayers and then polymerized. The interactions, in their opinion, could be due to electrostatic bonding with positively charged amino groups in macromolecules, as well through hydrogen bonds.

Iler in 1952 studied in detail the precipitation of gelatin with silica sol [175]. The experiments were made at pH 2, at which the condensation reaction proceeds at the lowest rate, which did not complicate revealing the features of the interaction with the protein. Studying the effects of various substances that inhibit the formation of hydrogen bonds allowed Iler to gain an insight into the mechanism of the processes. He suggested that polysilicic acids bind to macromolecules through hydrogen bonds and also act as a cross-linking agent.

The first attempt to immobilize enzymes was made by Dickey in 1955 [176]. He used a silica prepared from sodium metasilicate. It failed to adsorb trypsin, while adsorbed urease and catalase showed low activity for only one day.

Johnson and Whateley in 1971 made important adjustments to the Dickey immobilization procedure Johnson and Whateley in 1971 made important adjustments to the Dickey immobilization procedure [177]. They initially prepared a silica sol, added trypsin to the solution, and then carried out a sol–gel transition, as a result of which the enzyme was incorporated in the silica matrix. It was not washed out and exhibited esterase activity towards N-α-benzoyl-L-arginine ethyl ester hydrochloride throughout the 72 days of testing, retaining 90% of its original activity at the end of testing.

The method proposed by Johnson and Whateley [177] has merits that compare well with approaches developed much later, but it has not attracted the attention of researchers. The lack of interest may be due to publication in a colloid journal, which is not among those read by biochemists.

In an article published by Venton et al. in 1984 [178], immobilization of antiprogesterone antiserum was carried out by the conventional sol–gel method using TEOS in combination with a precursor containing a 3-aminopropyl group. It was emphasized that the alcohol released during hydrolysis had a detrimental effect on proteins, but in their case the effect was not so dramatic. Antiprogesterone antibodies retained ~50% of their original activity after immobilization. The authors mentioned the great potential of the sol–gel method for various applications, but the article, like the previous one, did not attract noticeable attention.

Glad et al. confirmed the results of previous work by immobilizing glucose oxidase and radish peroxidase [179]. They also showed the feasibility of the method in the case of trypsin and alkaline phosphatase. The authors used various precursors and their combinations, as well as a combination with commercial silica microparticles.

The feasibility of the sol–gel method for the immobilization of living cells in the case of yeast cells of *Saccharomyces cerevisiae* was first demonstrated by Carturan and co-workers [180]. They initially used an alcohol solution of TEOS, which, due to poor biocompatibility, was then replaced by a commercial solution of SiO_2_ sol [181]. Immobilized yeast cells showed activity towards sucrose an order of magnitude higher than the original culture in solution. It is important that it remained at the same level for 15 months [182].

Direct interest in the immobilization of biomaterials by sol–gel chemistry has appeared since the early 1990s of the last century after the publication of the article by Broun et al. [183]. The authors took alkaline phosphatase, undertaking, according to their statement, all necessary measures to reduce the denaturation of the enzyme. This made it possible to obtain an active biocatalyst, somewhat inferior in activity to the original phosphatase but preserved better when kept in an aqueous solution (2 months) and high temperature (70 °C). This gave impetus to their own systematic studying on the preparation of biocatalysts, including entrapment of biopolymers into silica matrix as well as to other teams [49,74,85,181,184,185,186,187,188,189,190,191,192,193,194,195,196,197]. A large body of results was summarized and discussed in numerous review articles (see, e.g., Refs. [7,20,26,27,28,29,30,42,44,86,160,198,199,200,201,202,203,204,205,206,207,208,209].

## 5. Advantages of Sol–Gel Immobilization

The first works on the entrapment of biomaterial into a silica matrix using the sol–gel chemistry method revealed a number of important advantages of the approach, which was the reason for increased attention and its rapid development [5,6,7,9,10,14,17,20,21,26,28,30,50,52,79,160,200,205,210,211,212,213,214,215,216,217,218,219]. Worthy of mention are the following:

- Control of the composition, structure, porosity and surface area of the silica matrix;
- A variety of forms—monoliths, films, (nano) particles, capsules, fibrils, powders;
- Modification of the silica surface by attaching functional groups, organic substances and polymers;
- Available cheap commercial precursors;
- Thermal and chemical resistance of SiO_2_;
- Optical transparency of the silica matrix;
- Protection from toxic chemicals, UV radiation and microorganisms;
- Increasing the long-term and thermal stability of the immobilized biomaterial in sol–gel matrix;
- Simple, readily available, and cheap equipment that does not require special precautions.

The noted advantages have made silica materials synthesized by sol–gel chemistry one of the most popular for the immobilization of biological material. However, the approach is not devoid of certain disadvantages, as mentioned in Section 2.4. Therefore, numerous attempts are being made to improve sol–gel synthesis, and approximate the conditions and compatibility to biosilicification in living systems, i.e., make it as biomimetic as possible. The main approaches proposed to date are considered below.

## 6. Biomimetic Approaches

### 6.1. Two-Stage Immobilization

**Figure 6:**
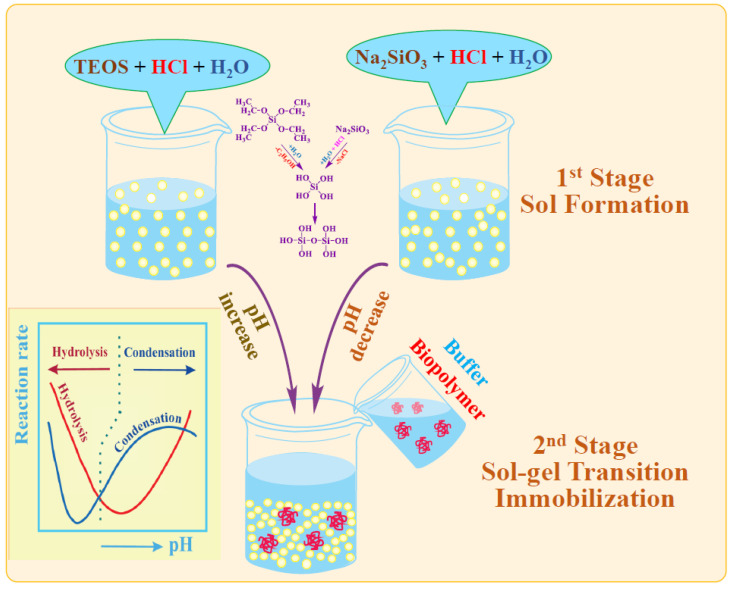
Schematic presentation of two-stage immobilization. Insert: kinetic of alkoxysilanes hydrolysis and condensation versus pH.

Immobilization via two stages was first carried out in one of the pioneering works in 1971 [177]. The aim was to prevent the denaturation of trypsin that occurs in an acidic solution, which the authors succeeded in achieving. Currently, the method has become quite widespread for incorporating enzymes and cells into a silica matrix while maintaining their functional properties. There are two versions (Figure 6). The most common approach involves pre-hydrolysis of the alkoxysilanes in an aqueous solution with a small amount of added acid—usually HCl. Sometime after obtaining the sol, a buffer solution without or containing proteins, enzymes, DNA, or cells is introduced into the reaction media. Its addition shifts the pH to a neutral region, in which condensation reactions (5) and (6) are sharply accelerated, which leads to a sol–gel transition and the formation of a silica matrix with the entrapped biomaterial.

The second, less popular approach is based on the use of sodium metasilicate. Its solution is highly alkaline and unsuitable for biomaterial. As described in Section 2.2, the sol–gel process is initiated by shifting the pH to the acidic side by adding an acid (Figure 6). There is a similar formation of silica sol. The second stage consists of adding the buffered solution with biomaterial.

The two-stage scheme is based on the difference in the dependence of the kinetics of hydrolysis and condensation reactions on the pH of the aqueous solution. This is shown schematically in Figure 6 (insert). Hydrolysis occurs at a high rate in the acidic region, in which condensation reactions are greatly inhibited. Therefore, the polymerization of the resulting silicic acid is limited to the formation of oligomeric products in the form of sol particles. A shift in pH to the neutral or slightly alkaline side triggers an exponential acceleration of condensation reactions, which leads to the cross-linking of sol nanoparticles into a three-dimensional network structure, in which enzymes and cells are engaged.

The main advantage of the two-stage method is the elimination of the negative impact of an acidic or alkaline media on the biomaterial; however, during the hydrolysis of alkoxides, alcohol is released, which is left in the solution. Its amount can be very significant, reaching 35% [220]. Many proteins are denatured at this ethanol or methanol content, and cells die [86,88,202]. The situation is aggravated by the addition of an organic solvent—usually an alcohol—to improve the solubility of TEOS or TMOS in the reaction mixture. It was suggested not to add them in [66,85,86]. After introducing alkoxysilane, the authors placed the separated mixture in an ultrasonic bath. Dispersing the alkoxysilane accelerated the hydrolysis and the formation of a homogeneous sol dispersionю.

Alcohol released after hydrolysis of the precursor was first removed during the synthesis of ordered mesoporous materials in order to eliminate phase separation of liquid crystalline systems [221]. To prevent the death of bacteria, it was evaporated from the solution by boiling it [222]. The method used, however, does not completely eliminate alcohol. It is more rational to use a rotary evaporator [223,224,225]. Removal is faster, under milder conditions including vacuum (30 mbar) and low temperature (48 °C), allowing for more complete alcohol removal [226,227,228]. The method was applied in some works, but it was not widely used due to its labor consumption, which significantly complicates biomaterial immobilization.

The original version of two-stage immobilization was proposed by Zhou et al. [219]. They prepared a solution of silica sol with whole blood cells, which was frozen at −80 °C. It was found that mineralization took place at such a low temperature and in a frozen state. The aging test, the kinetics of DNA degradation, as well as testing of resistance to UV irradiation and aggressive radical oxygen species after thawing, showed that the immobilized cells were preserved. The proposed method, according to the authors, allows for cost-effective long-term preservation of DNA. It was called cryosilicification.

Immobilization by means of a two-stage scheme has been applied in many laboratories, showing its effectiveness. It was successfully used to entrap alkaline and acid phosphatase [217,222,229], bacteriorhodopsin [74], butyrylcholinesterase [218], cholesterol oxidase and cholesterol esterase [230], cyclodextrin [231], cytochrome c [49,232,233,234], DNA [219,235], glucose-oxidase [184,236,237], horseradish peroxidase [87,232,238,239,240], lipase [157,241], lysozyme [82,83,242,243], myoglobin [49,242], RNA aptamers [66] tryptophan [244], tyrosinase [239]. In addition, the method has been used to immobilize bacteria [88,196,222,224,225,245,246], microalgae [44,227], and viruses [228].

The bottleneck of the two-stage method is the fabrication of the sol. It cannot be prepared in advance and stored for a long time. It must be consumed within 24 h, since if the shelf life is extended, gelation will occur, i.e., an uncontrolled sol–gel transition [77].

A further disadvantage is the presence of significant amounts of alcohol released during the hydrolysis of alkoxysilane (reaction 4). It denatures proteins and causes cell lysis. If alcohol has a pronounced effect, leading to the loss of most of the functional properties, then in this case its removal is required, which greatly complicates the technique due to the inclusion of additional procedures, making it much more complex.

### 6.2. Colloidal Silica

Liu and Chen proposed an alcohol-free process in which they excluded pre-hydrolysis of the alkoxysilanes by taking a solution of dispersion of silica nanoparticles in the form of commercial Ludox [247]. Diatom and nanoparticles obtained by the pyrolysis of rice husk are appropriate as well [248]. Since dispersions, in particular, Ludox, are stabilized against flocculation, cross-linking and formation of a three-dimensional silica matrix of nanoparticles were achieved by the addition of sodium metasilicate. Aqueous mixtures were prepared at an optimal pH of 6.2–8.2 for proteins (cytochrome c, catalase, myoglobin, and hemoglobin), to which they were added. After gelation, a bionanocomposite was formed with entrapped biomacromolecules that retained their functional properties. The use of a ready-made dispersion of SiO_2_ nanoparticles makes it possible to reduce the concentration of Na_2_SiO_3_ and, accordingly, the amount of sodium cations in the system.

A silica matrix from a commercial Ludox dispersion can be formed without the use of sodium metasilicate. This is achieved by concentrating the system—partially removing water. In the course of the process, nanoparticles come closer together and flocculate, forming a three-dimensional network [30]. The pre-added biomaterial is brought into the inorganic matrix.

The method can be applied for the immobilization of living cells, which are most sensitive to the process conditions [249]. It has been used to include bacteria *E. coli* and *Bacillus subtilis* spores [88,212,250], as well as plant cell culture (from BY2 tobacco calli and from carrot phloematic tissue) [251]. To reduce the negative impact of the process and prevent direct contact of silica with cells, gelatin was added to the reaction mixture [249]. Bacteria and plant cells in [250,251] were isolated in calcium alginate capsules, on the surface of which a silica shell was formed. In [252], yeast alcohol dehydrogenase was first placed in k-carrageenan capsules, the surface of which was coated with chitosan, and then with silica. The method is applicable in combination with both sodium metasilicate and alkoxides, some amount of which are replaced by silica nanoparticles.

Using the methods discussed above makes it possible to reduce the alcohol content released during the hydrolysis of alkoxides. However, a significant amount of it is still left in the solution. Ferrer et al. suggested to remove it completely, thus neutralizing the negative effect [223]. Removal was performed by means of a rotary evaporator.

### 6.3. Chemical Vapor Deposition

Silicon alkoxides are volatile matter. This property was first used in 1942 in the processing of textiles, paper, and wood [253] and later applied to the mineralization of biomaterial [33]. Alkoxides are supplied with a stream of moist air, in which their partial hydrolysis occurs. When silicic acid falls on the surface of an object, it enters into a condensation reaction, forming a silica shell. The method was mainly used for the immobilization of cells pre-deposited as a layer on a solid substrate [5,254]. A combination of methods is possible. The cell suspension in [255] was first combined with SiO_2_ sol and then treated in alkoxide vapor. Liver cells rat hepatocytes in [256] were bound in a collagen gel. In all cases, cell cultures maintained viability for a long time [254,255].

The advantage of chemical vapor deposition is its simplicity [33]. Coating by silica does not require complex equipment or large quantities of reagents. Post-treatment is minimal since the silica coating is obtained almost ready-made. No acid or alkali is used, and most of the ethanol or methanol released is carried away with the air flow. Disadvantages include strict control over vapor pressure and process temperature. In addition, the coating is formed heterogeneous, with defects [33].

### 6.4. Glycerol-Containing Silanes

The use of silicon esters with glycerol instead of alkoxides has attracted considerable attention over the past 20 years. It is explained by the ability of glycerol, along with sugar and amino acids, to significantly increase the thermal stability and bioactivity of enzymes [257,258]. Such substances are called osmolytes. Glycerol-containing silanes were first synthesized at the beginning of the last century—in 1916 [259]. Knorr and Weyland used a one-step transesterification reaction to replace the ethanol residues in TEOS with glycerol. The patent suggests that compounds with two and four glycerol residues, as well as an oligomeric product, were synthesized. The first two compounds were soluble in water, while the fourth was insoluble. Sol–gel processes have not been studied, but the authors proposed using the synthesized substances to treat tuberculosis, presumably considering them harmless.

Significant interest in glycerol-containing precursors appeared in the 1930s–50s of the last century. It was explained by their fairly simple synthesis, proposed in [259], as well as their use as intermediates for the production of silicones [260]. Their solubility in water was especially noted. Biomimetic applications were not considered at that time.

Gill and Ballesteros [261] were the first to use a glycerol-containing precursor for biomaterial immobilization. They synthesized not an individual substance, but an oligomeric product called poly(glyceryl silicate). The method consisted of partial (up to 50–75%) hydrolysis of TMOS in an alcoholic solution of HCl and subsequent polymerization of the product to oligomers [-Si(OCH_3_)_2_-O-]_n_ in which the alcohol residues were replaced by glycerol using a transesterification reaction catalyzed by hydrochloric acid or poly(antimony(III) ethylene glycoxide). The synthesized poly(glyceryl silicate) SiO_1.2_Glc_0.8_ was a solid compound, readily soluble in water. Gelation as a result of the sol–gel transition occurred after a few minutes in a neutral aqueous solution without the addition of a catalyst and organic co-solvent. Gill and Ballesteros were able to immobilize a large number of different enzymes and cell cultures in one step simply by admixing a precursor into their solution [7,203,261]. In the immobilized state, cells and a number of enzymes retained more than 90% of their initial activity. Comparison with TMOS and poly(silicic acid), obtained by polymerization of silicic acid, showed better biocompatibility of poly(glyceryl silicate) with the biomaterial. In the first two cases, activity decreased to 11–76%, and in glycerinated silica—to 83–98% [261].

Studies with poly(glyceryl silicate) were not continued. The reason, evidently, is that the precursor is not an individual compound, but a mixture of linear, branched, and/or cyclic derivatives [7]. Their reproducible synthesis seems quite problematic.

Khonina et al. proposed an alternative to the precursor, also synthesized by replacing ethanol in TEOS with glycerol HOCH_2_CH(OH)CH_2_OH, but in its large excess [262,263]. The authors argued that they obtained Si(C_3_H_7_O_3_)_4_ × 10C_3_H_8_O_3_ with a yield of 99%. Synthesis with the same amounts and concentrations of reagents as in the articles by Khonina et al. was repeated in [264]. A detailed study using ^29^Si NMR spectroscopy showed that the product is not an individual substance. It is rather a mixture of different isomers, including cyclics, potential dimers, and other oligomers.

Attempts to synthesize tetraglycerol orthosilicate (TGOS) or tetrakis(2,3-dihydroxypropyl) orthosilicate as an individual substance without adding excess glycerol have been made by a number of researchers. This was performed in the previously mentioned 1916 patent [259], and then in the 2003 patent [93] through a similar transesterification of alcohol residues to glycerol in TEOS or TMOS. The product yield was 72%. When a catalyst was used in [265], it increased to 97%. The yield in the synthesis, repeated in acetonitrile instead of mixing stoichiometric amounts of glycerol with TEOS or TMOS, did not exceed 75%. Repeating the synthesis with a catalyst in article [81] did not bring about the formation of TGOS in the form of an individual substance. The authors were unable to isolate it, suggesting that the product is most likely to be a mixture of various glycerol esters.

It should be noted that Breenan et al., in a patent [93], described the synthesis of three individual glycerol-containing precursors: mono-, di- and TGOS. Their preference was given to diglycerylsilane. Its synthesis is described repeatedly in patents [93,259] and articles [75,266]. It is characterized by good reproducibility and high yield, approaching 100%. This glycerol-containing silane, unlike others, can be isolated as an individual substance. A comparison of the activities of dihydrofolate reductase, cyclooxygenase-2, and γ-glutamyl transpeptidase immobilized with TEOS and diglycerylsilane in [220] showed that it was significantly higher in the latter case. The advantages of glycerol-containing precursors also include less shrinkage of silicas during aging. For example, the reduction in its size within 100 h after the sol–gel synthesis in the case of diglycerylsilane was 17%, and in the case of TEOS, 29% [93].

Breenan’s group, after patenting, used only diglycerylsilane in a series of subsequent papers [51,75,266,267,268,269,270,271,272], which in itself is indicative. Later, apparently, they did away with it, switching to two-stage immobilization [66,273,274]. Although adequate biocompatibility of the precursor and the conditions of the sol–gel process has been shown, it was not pronounced in a number of cases [9]. In particular, a significant decrease in the activity of immobilized horseradish peroxidase was observed in [65]. The authors associated the negative effect with glycerol, ca. 20% of which was released during hydrolysis.

Good compatibility with biopolymers and cell cultures is an undoubted advantage of glycerol-containing silanes [5,7,52,81,93,203,256,261,275], however, they are not widely used in biomimetic research. This may be due to a number of limitations.

- Individual substances. Except for the diglyceryl derivative, silanes are not isolated as individual substances. Poly(glyceryl silicate) and TGOS, even in excess of glycerol, refer to mixtures of various glycerol esters [7,81,264,276]. Accordingly, this affects the reproducibility of their synthesis and sol–gel processes.
- Inhibition of sol–gel processes. Khonina et al. pointed out [263,275] that glycerol has an inhibitory effect on hydrolysis and condensation reactions, slowing down the process as a whole. In the case of diglycerylsilane, a significant slowdown in the condensation reaction was observed in [75,277]. To hasten it, Breenan et al. used, for example, human serum albumin acting as a catalyst for silica formation. The inhibition effect is particularly pronounced in the approach of Khonina et al. who used in actuality a solution of the precursor in a 10-fold excess of glycerol (Si(C_3_H_7_O_3_)_4_ × 10C_3_H_8_O_3_). Therefore, the authors had to resort to various measures to speed up the sol–gel processes. In particular, the precursor was introduced into a strongly acidic solution (pH 1) and heated to 80 °C [262]. Under such conditions, biomimetic immobilization of biomaterial during the synthesis of a silica matrix is not possible. There is no need for heating, as determined from a study of the effects of various inorganic salts if potassium fluoride is added. KF plays the role of a nucleophilic catalyst, which is well-known in sol–gel chemistry [37,38,48].
- High viscosity. Glycerol is a highly viscous solvent. Its addition sharply increases the viscosity of aqueous solutions. This poses certain experimental difficulties in the form of slowing down sol–gel processes and complicating their regulation. In addition, the diffusion of substrates and the removal of reaction products slow down, which is reflected in a decrease in enzyme activity [65].
- Solid matter. Glycerol-containing precursors are solid substances, the work with which require additional actions to stimulate dissolution. In particular, diglycerylsilane in [277] was pre-crushed into a powder, which dissolved faster. Accordingly, this accelerated the sol–gel processes. In addition, a similar effect was achieved by ultrasonic treatment of solutions [65,75,272].

### 6.5. Polyethylene Glycol-Containing Silanes

A study of lipase in the immobilized state in [241] revealed an increase in its activity after the addition of water-soluble polyethylene glycol. Improved compatibility of the silica matrix with cellular receptors after the addition of the polymer was repeatedly noted in [9,271,278,279]. These observations may have been stimulatory to synthesize a precursor in which the alcohol residues were replaced by polyethylene glycol HO[CH_2_CH_2_O]_9_H or PEG400 [275]. Precursor was used for the mineralization of such polysaccharides as chitosan, hydroxyethyl cellulose and xanthan gum [280]. It was taken at a concentration of 99.0 wt.% due to precipitation in water at a lower content [280]. This mixture was not convenient for polysaccharides. In particular, chitosan precipitated at a concentration of 0.5 wt.% due to poor solubility in this solution. Furthermore, the formation of silica was slow due to steric restrictions (no data available). To accelerate the processes, the syntheses were performed at 80 °C. As in the case of the Si(C_3_H_7_O_3_)_4_ × 10 C_3_H_8_O_3_ considered in the previous section, the conditions cannot be believed to be suitable to the biomaterial. High temperature is crucial for proteins and living cells, i.e., the proposed method cannot be fully classified as biomimetic. Therefore, heating was not used when the methylotrophic yeast *Ogataea polymorpha* was immobilized in [281]. Khonina et al. applied a catalyst (sodium fluoride) in this instance.

### 6.6. Sugar-Containing Silanes

A Brennan et al., taking into account that osmolytes, along with glycerol, include sugar and amino acids [257,258], synthesized precursors with glucose (gluconamidyltriethoxysilane or N-(3-triethoxysilylpropyl)gluconamide) and maltose (maltonamidyltriethoxysilane), which were covalently attached to (aminopropyl)triethoxysilane [282]. A study of silica formed using them, as well as their mixtures with diglycerylsilane and TEOS, showed that syneresis was 15, 70, and 85% *v*/*v*, respectively. Gluconamidyltriethoxysilane has shown good protein compatibility [9,282]. Its mixing with diglycerylsilane and TEOS led to a significant improvement in the properties of the latter [51,272,282]. In particular, the syneresis of silica formed from diglycerylsilane was 54%, and in the case of its mixture with sugar-containing silane in a ratio of 5:1—13% [51]. Human serum albumin and *Escherichia coli* retained their activity better in a silica matrix formed from diglycerylsilane and TEOS with the addition of 10% gluconamidyltriethoxysilane [65,224]. A detailed study of the properties of albumin showed that immobilization made a notable contribution to the retention of the native conformation, increased thermal stability, and improved ligand binding affinity [51]. The authors suggested that the gluconolactone residue forms a shell around biomacromolecules and bacteria, preventing their direct contact with the silica [9,224].

The disadvantages of a precursor with sugar residues include their synthesis and a fairly high price. When applied with TEOS, the alcohol released after hydrolysis has a negative effect on the immobilized biomaterial. In addition, the use of gluconamidyltriethoxysilane with diglycerylsilane, the negative aspects of which were noted above (Section 6.4), adds to the disadvantages.

## 7. Tetrakis(2-hydroxyethyl)silane (THEOS)

### 7.1. Synthesis

Silicon esters with ethylene glycol residues, in contrast to glycerol-containing silanes, have been proposed to be synthesized in two different ways. The main synthetic approach is to replace the ethyl or methyl alcohol residues, respectively, in TEOS or TMOS with ethylene glycol. It was first applied to the preparation of silanes with two methyl groups using dimethoxy- or diethoxydimethylsilane. Interest in them was caused by the demand for the production of silicones [260]. The synthesis of the precursor in article [283], in which it was first described, is represented by the reaction:
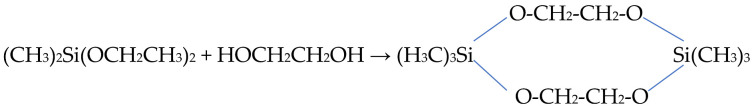
(7)

The synthesized product was a dimer. The monomer appears not to be the case. The synthesis in [284] was repeated in an excess of ethylene glycol, but the product was also a dimer. Its instability has been noted [283]. The dimer was synthesized as a solid compound containing a small amount of oily liquid. It increased over time. According to the authors, the oily liquid was a polymer, i.e., the silane was unstable, showing a tendency to be polymerized.

**Figure 7:**
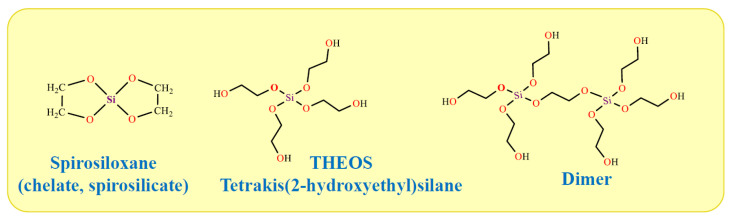
THEOS, spirosiloxane, and dimer synthesized in the course of the transesterification of TEOS with ethylene glycol.

The work in which THEOS was first synthesized is considered to be the article by Mehrotra and Narain [285]. However, the authors do not claim this. They performed the synthesis in benzene at the boiling point of the solvent in the presence of a p-toluene sulfonic acid as a catalyst, since the transesterification reaction proceeded very slowly [286]. Under these conditions, bis(ethane-1,2-diyldioxy)silane or spirosiloxane was obtained (Figure 7). It is also sometimes called chelate. Spirosiloxane differs essentially from the THEOS. The former is synthesized as an amorphous powder, and the latter as a colorless, transparent liquid. Doubts were expressed in [287] that Mehrotra and Narain were able to isolate it since the spirosiloxane easily polymerizes. The impossibility of its isolation was subsequently confirmed [288].

It is worth noting the publication of Mehrotra and Pant [289], in which silicon tetra-acetate, rather than alkoxide, was taken as the starting substance. The synthesis was conducted in benzene at room temperature. When ethylene glycol was added, heating of the reaction mixture was observed, since the exchange reaction of acetate residues for ethylene glycol is exothermic. The authors claimed to have synthesized THEOS, but the product was a solid, while THEOS is a liquid.

The publication of Kuznetsova and Belogolovina went unnoticed, but they synthesized THEOS for the first time in 1969 [290]. The synthesis was performed without the use of a solvent. A stoichiometric amount of ethylene glycol was added dropwise directly into liquid TEOS at 190 °C, distilling off the released ethanol. The product yield was 96.5%. The authors, by elemental analysis and the amount of alcohol distilled off, confirmed the preparation of THEOS, which was then used in the synthesis of polyurethane.

The attention of researchers to THEOS was attracted by the publications of Hoffmann and co-workers [291,292]. Although they stated that they carried out the synthesis according to the method of Mehrotra and Narain [285], in fact, it was quite close to that performed by Kuznetsova and Belogolovina [290]. The synthesized THEOS was a liquid rather than a solid spirosilicate. Importantly, Hoffmann et al. did not restrict themselves only to synthesis, but carried out a detailed study of sol–gel processes with THEOS, revealing its main advantages over TEOS.

A detailed study of the features of silane synthesis using ^29^Si-NMR spectroscopy was carried out by Hüsing and co-workers [276,293,294], the results of which are presented in detail in Ph.D. Thesis Hartmann in 2009 [295]. They were recently confirmed and supplemented by Bravo-Flores et al., who, in addition to ^29^Si-NMR, also used ^13^C- and ^1^H-NMR spectroscopy [296]. It has been established that during synthesis it is necessary to strictly control the temperature (140 °C) and exclude contact with air. The duration is ca. 15 h, but it can be halved [296]. THEOS is obtained in the form of a transparent, low-viscose liquid, which, according to our unpublished data, remains in this state for many years. If the synthesis conditions are violated, then a translucid viscous liquid or even a gel can form, which over time turns into a glassy mass. The diversity of products is due to the formation, along with THEOS, of chelates called spirosiloxane [297] or spirosilicate [298], as well as dimers and oligomers (Figure 7), which are in equilibrium with silane [276,293,294,295]. With strict control of the synthesis conditions, predominantly THEOS is obtained, the yield of which, according to the data of [295], based on thermogravimetric analysis, was 92%, and in [298] was found 88.6%. Interestingly, estimates of the amount of alcohol separated by distillation gave 99.4% [296].

A second approach that can be used to synthesize ethylene glycol derivatives of silicon is to react silica with ethylene glycol. The synthesis is carried out in excess of the boiling solvent (~200 °C) and in the presence of catalysts (organic amines, e.g., triethylenetetramine, and/or alkali hydroxides as co-catalyst). A detailed study using ^29^Si NMR, ^29^Si, ^13^C, and ^1^H MAS NMR, thermal gravimetric analysis, and mass spectrometry showed that THEOS and some amount of oligomers are mainly formed [297]. As the procedure is lengthened and ethylene glycol is removed from the reaction mixture, THEOS transforms into spirosiloxane Si(OCH_2_CH_2_OH)_2_ (Figure 7), which can become the main product. Its yield in [298] was >80%. Spirosiloxane was identified by a combination of physicochemical methods: FTIR, ^1^H, ^13^C, and ^29^Si NMR spectroscopies, thermal gravimetric analysis, and mass spectrometry.

The main advantage of the approach is the cheap and widespread raw material in the form of silica. The synthesis does not require expensive equipment, it is quite simple and environmentally friendly.

### 7.2. THEOS Advantages

Hoffmann et al. were the first to conduct a systematic study of THEOS, clearly demonstrating the significant advantages of the precursor over TEOS [291,292]. Firstly, the replacement of alcohol with ethylene glycol led to the appearance of hydroxyl groups on the surface of the molecule (Figure 7). This changed its polarity from hydrophobic to hydrophilic. Therefore, THEOS, unlike TEOS, is a substance that mixes well with water in any proportion. Accordingly, the introduction of an organic solvent to improve solubility is not required.

Of particular importance is the fact that sol–gel synthesis occurs in neutral solutions without the addition of acid or alkali. The hydrophilic ethylene glycol released during hydrolysis, as especially noted by Hoffmann et al. [291,292], does not affect or disturb the phase state of surfactants since it does not suppress the hydrophobic effect that determines their micellization. Due to the compatibility of silane with lyotropic surfactant phases, they remained unchanged after its introduction [291,292,299]. This was confirmed by Hüsing and co-workers, who were able to synthesize bimodal silicas with meso/macropores using an amphiphilic triblock copolymer as a template [300].

Sol–gel processes with a second ethylene glycol-containing precursor, spirosiloxane (Figure 7), were studied in detail in [301]. The presented data let us compare it with THEOS. These two precursors differ significantly in properties. Syntheses with spirosiloxane, as with TEOS, were carried out by adding a catalyst—acid or alkali. Increasing the amount of acid significantly reduced the gelling time, i.e., reactions accelerated in acidic solutions. In addition, heating of the reaction mixture was required. For example, at room temperature the process lasted more than 10 h, and at 40 °C—less than an hour [301].

The discussed results allow us to conclude that sol–gel synthesis with spirosiloxane requires more severe conditions than that with THEOS. They are detrimental for biomaterial. Therefore, this precursor cannot be recommended for biomimetic silicification.

The possibility of using THEOS for biomimetic syntheses was first demonstrated by Shchipunov. He formed bionanocomposites with carrageenan [302] and a number of polysaccharides [50,303], studying their properties in detail. Immobilization was carried out in neutral aqueous solutions. The introduction of organic solvents, catalysts, and heating of solutions were not required. Moreover, polysaccharides accelerated sol–gel processes, exerting a catalytic effect.

### 7.3. Mechanism of Sol–Gel Processes

**Figure 8:**
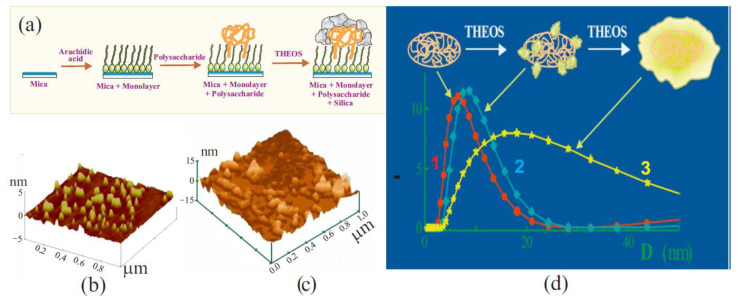
(**a**) Schematic drawing of experimental design to confirm silicification of polysaccharide by THEOS. Explanations are given in the text. (**b**) Atomic force microscopy image of a mica surface with a deposited monolayer of arachidic acid and hydrophobically modified cationic hydroxyethylcellulose adsorbed on it. (**c**) Image of the same surface after 40 s of treatment in a solution with 10 wt.% THEOS. (**d**) Schematic drawing explaining the results of silicification of bovine serum albumin in solutions with 0 (1), 0.5 (2), and 3.0 wt.% THEOS (3). The measurements were carried out using dynamic light scattering. Adapted from [304], with permission from Elsevier.

Verification of the compatibility of the precursor with biopolymers and the mineralization of biomacromolecules was obtained in model experiments. Schematic explanatory drawings and corresponding experimental data are shown in Figure 8.

The Langmuir–Blodgett technique was used to deposit a monolayer of arachidic acid on a mica surface with hydrocarbon chains oriented outward (Figure 8a) [304]. This led to hydrophobization of the surface. The next step was adsorbing hydrophobically modified cationic hydroxyethylcellulose with hydrocarbon chains -C_12_H_23_ attached to the macromolecule on the surface of the monolayer. The chains were embedded between the arachidic acid molecules, while the hydrophilic parts were outside. Silica does not precipitate on hydrophobic materials because of its hydrophilicity. Therefore, SiO_2_ appearance was expected only in the case of mineralization of biomacromolecules. Atomic force microscopy was used for visualization.

Experimental data obtained at different time intervals are shown in Figure 8b,c. At the initial stage, yellow spots, which are clusters of adsorbed biomacromolecules, can be seen on the surface of mica coated with a monolayer of arachidic acid (Figure 8b). The following image shows the surface after its treatment in THEOS solution within 40 s (Figure 8c). Clear changes are evident. The spots grew mostly in height. When the treatment was longer (not shown), the surface was completely covered with SiO_2_, due to their growth in the lateral plane. At the same time, noticeable amounts of silica did not have time to form in the bulk of the solution in which the polysaccharide was not added [304].

A recently published paper [296] presents data on the silicification of chitosan by THEOS. It was found by means of FTIR, ^29^Si and ^13^C MAS spectroscopy that silica precipitated on polysaccharide macromolecules. Bravo-Flores et al. revealed the formation of covalent bonds with the terminal hydroxyl groups attached to C6. This suggests the feasibility of a condensation reaction between ≡Si-OH and the hydroxyl group of the polysaccharide.

Another experimental verification of biomimetic mineralization of biopolymers was obtained with bovine serum albumin. The protein has a prolate ellipsoidal shape (a, b, b) with certain dimensions: a = 7.0 nm and b = 2.0 nm [305,306]. Their measurement provided a way of determining the precipitation of silica if there is the formation of SiO_2_ coating on the biomacromolecule surface. The results obtained by the dynamic light scattering method are shown in Figure 8d. The initial dimensions of the biomacromolecule (curve 1) correlated well with the literature data. The results of measurements in two neutral albumin solutions of the same concentration, to which 0.5 (curve 2) and 3.0 wt.% THEOS (curve 3) were added, are also presented. Silane was introduced in an amount that did not exceed the critical concentration for the onset of gelation of solutions (~5 wt.%). Measurements showed that the dimensions of the biomacromolecule in solutions with 0.5 and 3.0 wt.% THEOS increased, correspondingly, by 1–2 nm and by at least 2.5 times (Figure 8c). Other nanoparticles were absent in their solutions. This led to the conclusion that the preferential site for localization of sol–gel processes at neutral pH is albumin macromolecules [52].

**Figure 9:**
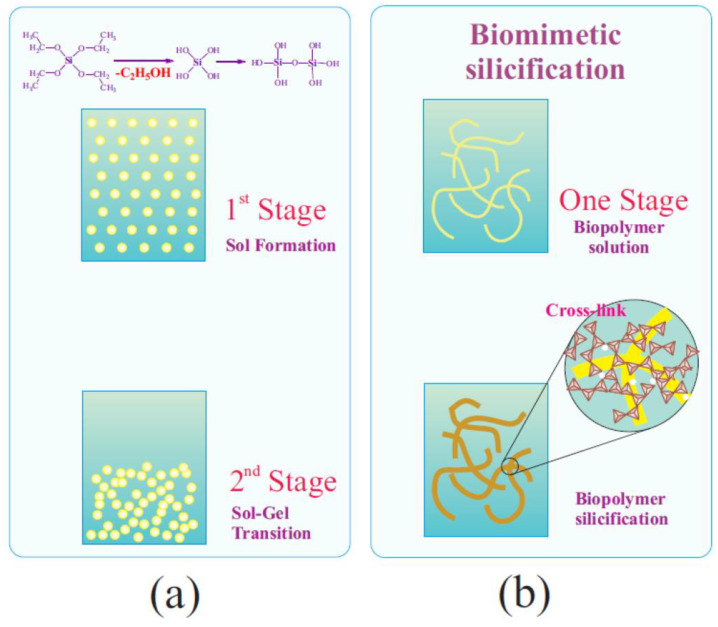
Schematic drawings of two-stage traditional sol–gel processing with TEOS (**a**) and one-stage biomimetic silicification using THEOS (**b**). Further details are discussed in the text.

The mineralization of biomacromolecules, rather than the formation of sol nanoparticles, as occurs in the case of the well-established sol–gel process, points to a fundamental difference in their mechanisms. Hydrolysis of TEOS or TMOS, which, due to their hydrophobicity (Figure 1) and lack of compatibility with the biomaterial, proceeds independently, leads to the formation of a sol, as shown schematically in Figure 9a. The formation of a three-dimensional network from it or silica matrix can only occur when the nanoparticles come into contact with each other (Figure 9b). Therefore, the sol–gel transition is always accompanied by a characteristic phenomenon called syneresis. It consists of phase separation into solid silica and an aqueous solution. As a result of syneresis, up to 70% of the solution can be separated [52,76].

Sol–gel processes with THEOS, as shown in [52,296,304,305], are localized predominantly on biomacromolecules. Silicification initially leads to the formation of separate small clusters, which merge due to further growth into a silica shell (Figure 8a and Figure 9b). Biomacromolecules, in contrast to separated sol nanoparticles, form a three-dimensional network in the bulk solution [307,308,309]. At the intersection points of the chains, crosslinks form from the generating SiO_2_ (Figure 9b). They mechanically lock the existing three-dimensional network of biomacromolecules, preventing its collapse [52]. As a result, the syneresis of bionanocomposites prepared using THEOS is either absent or manifested to a minimal extent [50,52,302,303].

### 7.4. THEOS versus TGOS and Diglycerylsilane

Silicon esters with ethylene glycol and glycerol have excellent biocompatibility, which has made them popular as precursors for biomimetic immobilization of biomaterial [52,262,264,266,276,296]. Precursors were compared in only work [81]. The authors correlated the features of sol–gel syntheses with silicatein A1 separated from the marine sponge *Latrunculia oparinae*. Quite a good similarity in the properties and biocompatibility of THEOS and TGOS was noted. It emerged most clearly from a comparison with TEOS. According to the authors of [81], TGOS does have some advantages over THEOS, which, in our opinion, are not so obvious. When various factors are also considered, then preference should still be given to THEOS.

THEOS is synthesized from commercially available TEOS or TMOS in one stage without the addition of catalysts and solvents. It is obtained in yields of up to 96–98% as an individual substance [52,276,291,293,295,296]. The only by-product alcohol is easily separated by distillation during synthesis. Silane is a low-viscose liquid, which facilitates mixing with aqueous solutions of biopolymers and cells, and, accordingly, performing sol–gel syntheses.

TGOS synthesis involves some difficulties. It has been suggested to synthesize it in a large excess of glycerol, in which a mixture of various glycerol esters is found, or in the form of oligomeric poly(glyceryl silicate) of uncertain composition [7,81,264,276]. An individual compound obtained in high yield is diglycerylsilane, but in distinction to THEOS, it is isolated in solid form. To introduce it into the sol–gel process, it was first crushed into powder [278] or the solutions were previously exposed to ultrasonic treatment [65,76,273]. Glycerol, released in the course of hydrolysis or present in the reaction mixture, is a highly viscous solvent that inhibits hydrolysis and condensation reactions, slowing down the overall process [76,264,276,278]. To speed them up, it is necessary to resort to various additional efforts [263].

The literature survey shows that THEOS has received wider recognition and is used by more research groups. The list of immobilized biomaterials is more extensive. It includes various polysaccharides, proteins, enzymes, polyphenols, and living cells, which has led to the development of various functional materials.

### 7.5. Applications of THEOS

THEOS as a precursor provides a variety of opportunities for the biomimetic sol–gel synthesis of bionanocomposites, the structure and properties of which are regulated by biopolymers that act as templates and also impart functionality inherent in living systems. The main avenues of investigation are considered in later sections.

#### 7.5.1. Hydrogels

The gelation of solutions with biopolymers is one of the valuable properties. Their hydrogels are widely applied in the food, cosmetic, and biomedical industries [310,311,312,313,314,315,316,317]. However, many of the polysaccharides do not have the ability to jellify solutions and can only increase the viscosity of aqueous solutions to a very limited extent [310,311,312,313,314,315,316,317]. The lack of gelling ability sharply limits the application and reduces the practical value of such well-known biopolymers as hyaluronate, chitosan and cyclodextrin. Therefore, widespread methods for imparting this ability to them are modification and cross-linking by chemical methods [314,317,318,319,320,321]. The aim is achieved, but at the same time, the biocompatibility of the resulting biomaterials sharply deteriorates, which does not allow expansion and may even limit its use.

**Figure 10:**
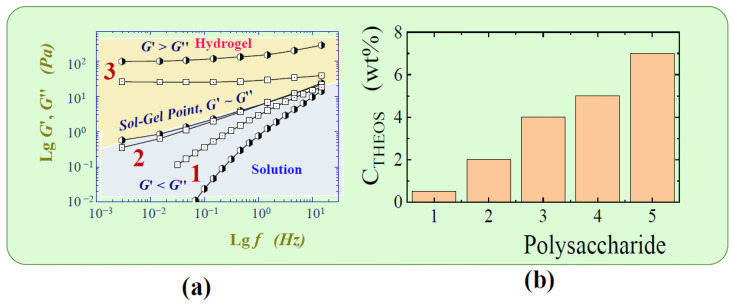
(**a**) The storage modulus G′ and loss modulus G″ against the oscillation frequency *f*. All solutions contained 1.5 wt.% cationic derivative of hydroxyethylcellulose and 0 (1), 0.5 (2) and 5 wt.% (3) of THEOS. (**b**) Concentrations of the sol–gel transition for cationic derivative of hydroxyethylcellulose (1), sodium hyaluronate (2), alpha- and beta-cyclodextrin (3), locust bean gum (4), chitosan (5). Adapted from [322], with permission from Elsevier.

It was suggested in [322] to use sol–gel chemistry to form hydrogels from polysaccharides that do not have this ability. The manner in which this task can be realized is shown by the example of a cationic derivative of hydroxyethylcellulose used in cosmetics [323]. In Figure 10a shear moduli are plotted as a function of the oscillation frequency for three polysaccharide solutions with three different THEOS concentrations. Three different states are clearly recognized from the rheological data. At the lowest content of THEOS, there is a solution, and at the highest, there is a hydrogel. In the first case, the storage modulus is less than the loss modulus in the whole frequency range, and in the second case, their position relative to each other has changed to the opposite. The transition from one state to another is characterized by a gel point [324], in which both modules are equal in magnitude and vary with frequency in the same way. It occurs in the case under consideration with the addition of only 0.5 wt.% THEOS. The gelation of the solution is due to cross-linking at the contact points of silica clusters (Figure 9b) formed during hydrolysis of THEOS on biomacromolecules.

The THEOS concentrations at which the transition to the gel state began for a number of polysaccharides [322], which are of great practical importance but are unable to form hydrogels, are shown as a diagram in Figure 10b. It can be seen that they vary from 0.5 to ~7 wt.%. This is indication of a significant dependence of the gel point on the nature of the biopolymer. It is important to note that the concentrations were small. Furthermore, the reaction products are only silica and ethylene glycol which are non-toxic substances. Their content does not exceed 5 wt.% in the maximum case. In view of the fact that synthetic amorphous silica is approved as a food additive by the United States Food and Drug Administration and European Food Safety Authority [12,17,46,47], hydrogels formed by silicification of polysaccharides may find wide application in areas in which non-toxicity and biocompatibility are a priority.

#### 7.5.2. Control of Morphology and Porosity

**Figure 11:**
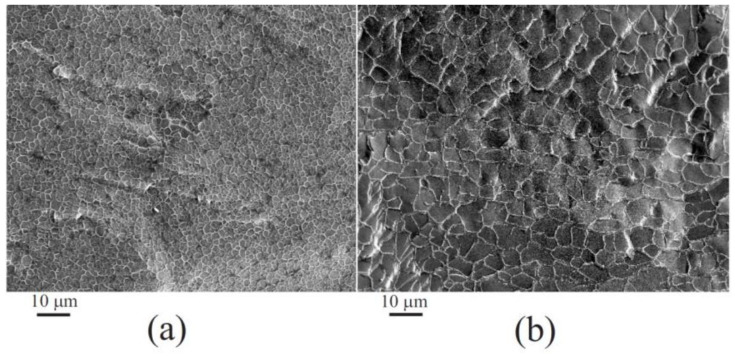
Scanning electron microscopy images of frozen hydrogels of cationic derivative of hydroxyethylcellulose (**a**) and chitosan (**b**). The concentrations of polysaccharides were 1.0 wt.% and THEOS 10 wt.%. Adapted from [303], with permission from American Chemical Society.

The distinctions in gelation concentrations and rheological properties noted in the previous section is a good indirect indication of differences in the three-dimensional network structure formed by the mineralized polysaccharides. It is borne out by pictures of samples prepared by adding 10 wt.% THEOS into solutions with 1 wt.% cationic derivative of hydroxyethylcellulose (a) and chitosan (b) in Figure 11 [303]. Their comparison reveals differences in the network structure. It is significantly denser in the former case than in the latter. This is in complete agreement with the rheological data (Figure 10b): the transition to the gel state in the presence of cationic derivative of hydroxyethylcellulose occurred at 0.5 wt.%, and in the sample with chitosan, at ~7 wt.% [322].

The structural features of macromolecules seem to be important for the differences. The cationic derivative of hydroxyethylcellulose is a graft biopolymer, while chitosan is linear. Branching creates additional opportunities for topological entanglements and the formation of a denser network structure.

A fundamentally different type of structure directing biomacromolecules is represented by cyclodextrins. They were studied in [325]. Cyclodextrins are formed by 6, 7, or 8 α-glucose residues, which are connected not in a linear chain, but in a closed, cyclic one [320,326]. The incorporation of α and β-cyclodextrins into the silica matrix was carried out in one step at room temperature and pH close to neutral [325]. In articles [327,328,329], in which TMOS was taken, two stages and preliminary removal of methyl alcohol were required to prevent the precipitation of polysaccharides. Cyclodextrins, acting as a template, catalyzed the sol–gel process with THEOS, causing the formation of a homogeneous microporous structure [325]. The pore diameter (0.6–0.8 nm) turned out to be somewhat smaller than the size of macromolecules (1.37 nm), which was explained by the shrinkage of the silica matrix during annealing (500 °C, 5–6 h), made to remove organic components from SiO_2_.

Published data shows that the biomimetic silicification of polysaccharides with THEOS opens up broad prospects for controlling the network structure, morphology, and porosity of SiO_2_, and, accordingly, the mechanical properties of bionanocomposites. This is performed by simply changing the biopolymer, varying its concentration, as well as the concentration of the precursor and their ratio. A number of articles are devoted to this problem [50,302,303,322,325,330].

#### 7.5.3. Proteins as Structure-Regulated Template

Proteins represent a very interesting tool for bottom-up regulation of the structure of the synthesized silica [52,139,331], because the conformation of biomacromolecules, and, accordingly, the secondary and tertiary structures depend on the experimental conditions. Even small changes in pH, temperature, and ionic strength of the solution, as well as the addition of salts, organic substances, and solvents, lead to significant rearrangements, characterized as denaturation [84,159,306,332]. In addition, proteins contain both positively and negatively charged groups, the density and location of which, as well as the ratio between them are changed when the experimental conditions are varied. They act as nucleation centers for oppositely charged substances, providing additional opportunities to control the structure of materials during silicification. It is also important to note that there is a wide variety and cheap sources of proteins. Many of them are characterized in sufficient detail, which simplifies the selection of a template. When using a compatible precursor, proteins, as established for the example of albumin, did not experience any significant changes, retaining their native structure [333], but they are able to regulate the structure of the silica. Experiments with horseradish peroxidase and myoglobin revealed in [139] that the effect of proteins was pronounced on SiO_2_ structure over length scales spanning five decades in size. Due to the fact that their secondary and tertiary structure can be precisely controlled in situ, they are of great interest in controlling the morphology, shape, and surface properties of silicas [331].

**Figure 12:**
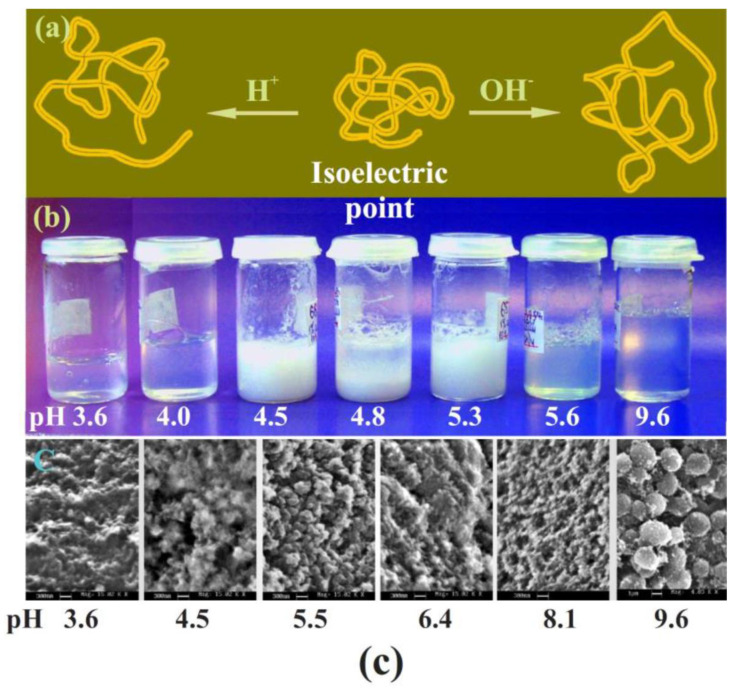
(**a**) Schematic drawing of change in protein conformation with the pH variation. (**b**) Pictures of vials with silica gel synthesized in bovine serum albumin solutions at various pH and (**c**) their scanning microscope images. Concentration THEOS was 10 wt.%. Adapted from [334], with permission from Elsevier.

An example is work [334], in which experiments were carried out with albumin, gelatin, and casein. It was shown that a change in their conformation by a simple pH shift on both sides of the isoelectric point (Figure 12a), leading to denaturation, can serve to finely regulate the structure of the silica. At the isoelectric point, the biomacromolecule has the most compact chain packing due to strong intramolecular electrostatic interactions between oppositely charged groups, the number of which is approximately equal to each other [84,159,306,332]. When the pH is shifted in one direction or another, the balance is disturbed. The greater the pH shift from the isoelectric point, the greater the number of positively or negatively charged groups begins to prevail. Electrostatic repulsion between similarly charged groups leads to a looser packing of chains (Figure 12a). The biomacromolecule increases in size (denatures). If sol–gel synthesis with THEOS is performed in albumin solutions with different pH values, as conducted in [334], silica with different optical properties is obtained (Figure 12b). This is due, as can be seen from scanning electron microscope images (Figure 12c) to significant changes in the morphology of the silica matrix. They were determined by conformational rearrangements of the protein macromolecule [52,334].

Interesting results were obtained in the article [335], in which the silicification of bovine serum albumin was made with small amounts of THEOS. The concentration was insufficient to gel the entire volume. The formation of silica clusters was predominantly localized on the surface of biomacromolecules. It is intriguing that crystalline microparticles of SiO_2_ in the form of triangular and rhombic dodecahedronal crystals were isolated by centrifugation upon completion of the experiment.

**Figure 13:**
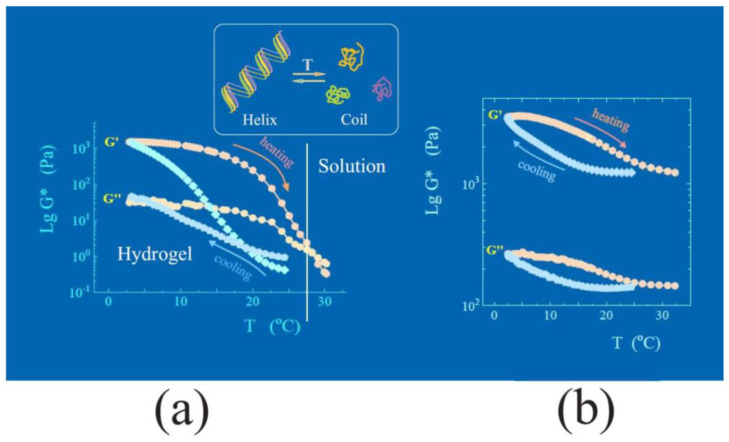
Insert: schematic presentation of helix to coil transition experienced by gelatin with temperature. (**a**) The storage G′ and loss G″ moduli against the temperature for hydrogels of 5 wt.% gelatin and (**b**) 5 wt.% gelatin and 10 wt.% THEOS. Measurements of the shear moduli were made at 1 Hz. Adapted from [334], with permission from Elsevier.

Another method of regulating the structure and mechanical properties of bionanocomposites was shown using gelatin [334]. This fibrillar protein is obtained by partial hydrolysis of collagen [336,337]. It retains a number of its properties. In particular, biomacromolecules in aqueous solutions are associated with the formation of double and triple helices, as schematically shown in Figure 13. The aqueous gelatin solution is in the gelling state. Its heating leads to melting, caused by the helix to coil transition, and cooling results in the jellifying owing to the association of biomacromolecules in helices. Both of these transitions are well determined using rheology. The results of the corresponding rheological measurements are shown in Figure 13a as a dependence of shear moduli on the temperature. At low temperatures, the storage modulus is greater than the loss modulus. When heated, the modules decrease, and when the melting temperature of the hydrogel is reached, which occurs at the helix to coil transition, the curves intersect each other. After that the loss modulus becomes greater than the storage modulus. Cooling restores the initial gelling state, since the transition is reversible and can be repeated many times.

Gelatin in the article [334] was biomimetically incorporated into a silica matrix by using THEOS. The change in the mechanical properties of the synthesized bionanocomposites is shown in Figure 13b. As seen, the modules change with temperature, but not so significant and without the intersection of the curves as in Figure 13a. This means that the protein undergoes some conformational rearrangements, but changes in the secondary and tertiary structure are not so significant as that in solution. The silica matrix has a stabilizing effect, evidently impeding the complete helix to coil transition. Protein in its turn has a noticeable effect on the mechanical properties of silica, although it is one of the most stable inorganic materials, having a very small coefficient of thermal expansion [69].

The given example shows how bioorganic and inorganic components can mutually influence each other, determining the structure and properties of hybrid materials. This is widespread in living nature.

Protein-regulated formation of silica coatings on the surface of materials has been demonstrated in [338]. The authors took recombinant silicatein, which was adsorbed on the surface of mica. It catalyzed sol–gel processes with THEOS, causing the precipitation of SiO_2_. The authors believe that this can be used to form ordered silica structures on various surfaces.

Great opportunities are provided by surfactants with amino acid residues. They have the property of self-organizing into a variety of unique structures, whereas amino acids catalyze THEOS hydrolysis and condensation reactions, causing their biomimetic silicification. In [339], fibrils were synthesized on a template formed from N-dodecanoyl-β-alanine. Sacamoto et al. [340] used a surfactant with a cholesterol residue, which self-organizes into various lyotropic liquid crystal phases. Thanks to the unique structural organization of the template, they were able to synthesize mesoporous silica with ribbon-like structures.

#### 7.5.4. Biocatalysts and Biosensors

Biocatalysts and biosensors are prepared by incorporating enzymes into a silica matrix. Since enzymes are proteins, their biomimetic silicification has much in common with what was discussed in the previous section.

**Figure 14:**
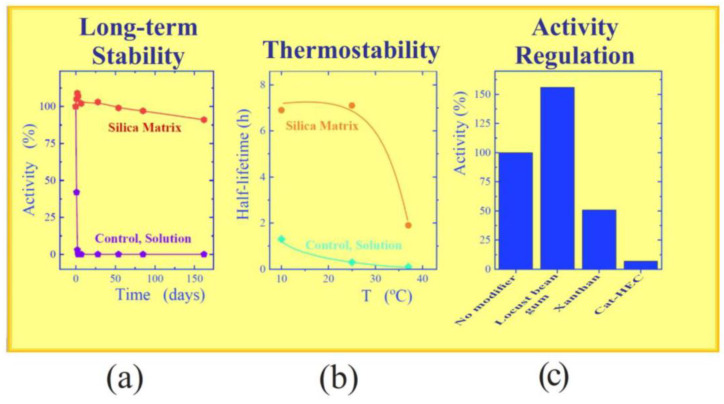
(**a**) Long-term stability and (**b**) of thermostability of 1→3-β-D d-glucanase L_o_. (**c**) Effect of polysaccharides on the 1→3-β-d-Glucanase L_IV_. Details are discussed in the text. Adapted from [342], with permission from Elsevier.

The feasibility of using THEOS for biomimetic immobilization of enzymes with complete retention of functional properties was first demonstrated in [341]. The results were confirmed in subsequent articles [342,343]. The main ones are presented in the form of plots in Figure 14.

The studies in the publications cited above were carried out with 1→3-β-d-Glucanase L_IV_, isolated from the marine mollusk *Spisula sacchalinensis* and marine bivalvia *S. Sacchalinensis*, as well as with 1→3-β-d-glucanase L_o_, isolated from marine bivalvia *Ch. Albidus*. In addition, α-galactosidase from the marine bacterium *Pseudoalteromonas* sp. was immobilized in the same manner. 1→3-β-d-glucanase L_o_ was characterized by increased instability. It denatured and lost activity at room temperature during the working day. This created great problems when studying the enzyme. After its entrapment in a silica matrix formed at an optimal pH value for the enzyme and a low temperature (3–5 °C), long-term stability increased sharply, as can be seen in Figure 14a. Immobilized 1→3-β-d-glucanase L_o_ retained activity for several months. The obtained result can be explained by the significantly increased thermal stability (Figure 14b). After immobilization, the enzyme retained its activity for some time even at 37 °C, and in solution, it was deactivated almost immediately.

Of special note is that the substrate for these enzymes was polysaccharides, i.e., high-molecular substances. However, they did not lose activity after immobilization, indicating their accessibility to the substrate. This is only possible with appropriate porosity of the silica matrix. The following results are noteworthy.

1→3-β-d-glucanases can catalyze both hydrolysis and glucanosyl transferase reactions, which proceed simultaneously and practically with almost equal efficiency [344,345]. The test showed complete retention of this ability in the immobilized state. Experiments were performed with the linear polysaccharide laminaran. It was hydrolyzed by ~50% after a 5-day enzymatic synthesis with immobilized 1→3-β-d-glucanase L_o_. In addition, among the reaction products was new branched 1,3;1,6-β-d-glucan with a molecular weight of 8 kDa, the yield of which relative to the initial linear laminaran reached almost 10% [342]. This means that the high molecular weight substrate had access to the enzyme immobilized in the silica matrix, and the reaction products could freely emerge into the surrounding solution. This is only possible with a silica matrix of high porosity.

The possibility of regulating the activity of immobilized enzymes by polysaccharides, which were together included in SiO_2_ in the course of the sol–gel synthesis, was examined in [341,342,343]. As obvious from the diagram in Figure 14c, their addition had a very noticeable effect on the functioning of 1→3-β-d-Glucanase L_IV_. Polysaccharides also influenced the activity of other enzymes studied, but the effects sometimes varied significantly. For example, locust bean gum in the case of 1→3-β-d-glucanase L_o_ led not to an increase, but to a decrease in enzymatic activity, i.e., the effect was individual in nature, which can be explained by differences in the secondary and tertiary structure of proteins.

A series of articles was devoted to the fabrication of electrochemical biosensors, which were based on the immobilization of horseradish peroxidase [346,347,348]. The use of chitosan and THEOS was also common. The polysaccharide, as was established previously [303,341,349], was introduced as a catalytic additive that accelerated sol–gel processes and promoted the immobilization of small amounts of the enzyme [333,346]. Unmodified chitosan was used only in [346]. In [347,348], the authors took carboxymethylchitosan and chitosan with attached β-cyclodextrin to incorporate, respectively, carbon nanotubes and polyaniline, serving to increase the electrical conductivity of the silica matrix. The use of THEOS allowed the enzyme activity to be held at a high level. The biosensors were characterized by good redox electroactivity towards hydrogen peroxide, a low detection limit, and a wide linear range.

#### 7.5.5. Immobilization of Living Cells

The entrapment of living cells in a silica matrix poses a great challenge due to the impact of various chemical and physical stresses, which can be very harmful and lead to their death [10,18,27,79,245,350]. Cell survival in an immobilized state is a major concern. The sol–gel method is one of the most popular due to a number of advantages mentioned in Section 5.

THEOS heretofore has been used for cell immobilization in only [351]. Experiments were conducted with the microalga *Porphyridium purpureum*. These photosynthetic microorganisms are highly sensitive to environmental pollution, which makes it possible to determine the appearance of even trace amounts of toxic organic and inorganic substances by their reaction [34,44,113,352,353,354,355].

The microalgae *Porphyridium purpureum* was immobilized in one step under conditions (pH, salinity, and temperature) that were maximally favorable [351]. Therefore, the cells remained alive after entrapment in the silica matrix. In addition, their thermal stability has increased. The response to pollution was determined by the fluorescence of chlorophyll-a and phycoerythrin, which are part of the photosynthetic system of microalgae. Some changes in their spectral characteristics were noted, which might indicate the influence of both the procedure itself and the resulting silica matrix. The period of functional activity of microalgae was rather short. The results signify that further optimization of the procedure is needed.

#### 7.5.6. Optical and Photonic Materials

**Figure 15:**
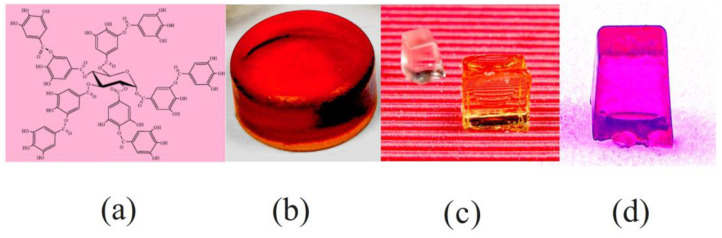
(**a**) Structural formula of tannic acid. (**b**,**c**) Pictures of the tannic acid–SiO_2_ bionanocomposites synthesized in the form of a disk (**b**) and cubes (**c**). Left sample in (**c**) without tannic acid, which was washed off by water. (**d**) The same sample as in (**c**) after the synthesis of gold nanoparticles. Adapted from [357], with permission from MDPI.

One of the fundamental properties of silica is optical transparency. Sol–gel technology is widely used to produce glasses [48,67]. A good example of an optically transparent material synthesized biomimetically is silica obtained with tannic acid (Figure 15a–c) [356,357]. When performing the synthesis with TEOS in an alkaline medium, a shapeless, opaque powder was formed [358]. THEOS enabled the process to be conducted at neutral pH, excluding precipitation and polyphenol oxidation. The synthesized, optically transparent material had a color caused by tannic acid (Figure 15b,c). The entrapped polyphenol retained its property as a reducing agent. This has been used to synthesize nanosized particles of silver and gold [357]. Their formation in situ in the pores of the silica matrix, in which tannic acid was left, led to a color change due to surface plasmon resonance characteristic of noble metal nanoparticles (Figure 15d) [359,360].

Silicas, including glasses, which are synthesized by sol–gel chemistry, do not have optical nonlinear properties. Non-linearity is imparted by special processing. The silica is first fused in an Ar atmosphere or in a vacuum at 1600–1800 °C, and then subjected to proton implantation, electron beam irradiation, UV poling, corona discharge-assisted poling, or thermal poling [361]. In the latter case, the sample is exposed to continuous high voltage (8–12 kV) at a temperature of ~280 °C [362]. The second-order nonlinearity appears only in a thin layer on the anode side. Meanwhile, the spicules of glass sponges *Hyalonema sieboldi* and *Pheronema* sp., which are formed at low environmental temperatures, exhibit optical nonlinearity. It is even higher than that of quartz fibers [363].

**Figure 16:**
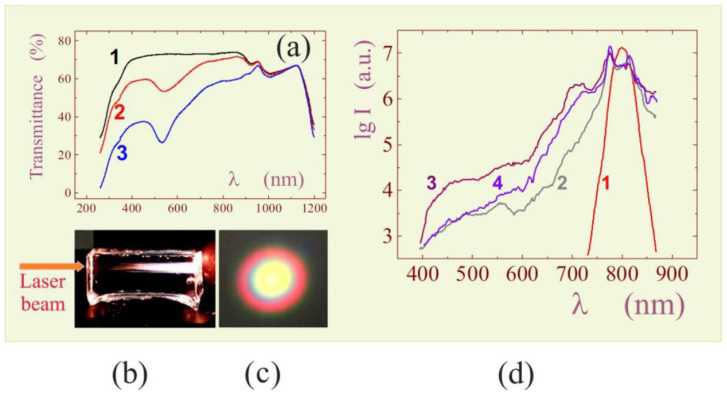
(**a**) Transmittance spectra of bionanocomposites synthesized in solutions with 1 wt.% hyperbranched polyglycidol by adding 50 wt.% THEOS in the absence (1) and presence of 2.0 × 10^−5^ (2) and 3.8 × 10^−5^ M HAuCl_4_ (3). (**b**) Emitted white light consisting of filaments generated in silica matrix of 14 mm in length and (**c**) conical beam outgoing from it. (**d**) Spectra of laser beam (1) and emitted light from hyperbranched polyglycidol–silica bionanocomposites synthesized in the absence (2) and presence of 2.0 × 10^−5^ (3) and 3.8 × 10^−5^ M HAuCl_4_ in solution (4). Adapted from [364].

A study of bionanocomposites containing sodium hyaluronate, sodium alginate, or xanthan, which were synthesized biomimetically at room temperature using THEOS, revealed optical nonlinearity when passing femtosecond laser pulses [363]. Evaluation showed that nonlinear indexes n_2_, equal to 29 × 10^−14^ cm^2^/W, was significantly higher than that of fused silica. The highest level of well-reproducible nonlinearity was found in silica with sodium hyaluronate. Comparable results were found in the case of bionanocomposites with hyperbranched polyglycidols as well [364]. They are dendritic macromolecules with random branch-on-branch topology. Their aliphatic polyether backbones, like polysaccharides, contain multiple terminal hydroxyl groups [365], on which silica precipitation occurs. The synthesized bionanocomposites were optically transparent (curve 1, Figure 16a). The introduction of gold nanoparticles led to a slight decrease in optical transmittance due to the appearance of surface plasmon resonance, the magnitude of which depended on their content (curves 2 and 3, Figure 16a).

The study of nonlinear optical properties of bionanocomposites with sodium hyaluronate and hyperbranched polyglycidols revealed a set of new properties, of which high third-order optical nonlinearity should be pointed out [366,367,368]. It was almost three orders of magnitude greater than that of fused quartz. A striking manifestation of the mentioned nonlinearity was supercontinuum generation. The phenomenon consists of the appearance of a bright white glow from multiple distinct filaments in the sample bulk when passing a series of femtosecond laser pulses with a duration of 40 fs and a frequency of 100 Hz. As an illustration, there is an image in Figure 16b. Next to it is a picture of the emerging beam, taken in a plane perpendicular to its axis (Figure 16c). One can see a bright white core surrounded by spectral colors. The white glow is due to a sharp expansion of the narrow spectral characteristic of the laser beam centered at 800 nm over the entire spectral range of visible light. The corresponding spectra 1 and 2 are shown in Figure 16d.

It is important that the optical nonlinearity can be controlled, and, accordingly, the supercontinuum phenomenon [366,367,368]. The spectra of samples containing hyperbranched polyglycidol and gold nanoparticles are shown in Figure 16d. The addition of the latter led to a significant increase in white emission, as can be seen from a comparison of spectra 2 and 3. The supercontinuum intensity is approximately an order of magnitude higher in the sample with trace amounts of gold nanoparticles (curve 3) compared to the bionanocomposite that does not contain them (curve 2). The difference holds at the same level in almost the entire visible spectral range from 420 to 720 nm. A comparison of spectra 3 and 4 shows that the effect depends on the content of Au nanoparticles. An increase in the intensity of supercontinuum generation was observed until a certain critical concentration of nanoparticles was reached. When it was exceeded, there was a sharp decay, which was explained by an increase in surface plasmon resonance related to nanoparticles (Figure 16a) [364].

The considered regulation of optical nonlinearity by means of the added gold nanoparticles was also offered by the exchange for quantum dots [368,369]. Their entrapment in a silica matrix synthesized using THEOS, the fabrication and properties of fluorescent bionanocomposites are described in a series of publications [370,371,372,373].

#### 7.5.7. Bionanocomposites with Fluorescent, Sensorial and Catalytic Properties

Fluorescent materials with quantum dots are mentioned above. They were also formed biomimetically by incorporating the organic luminophore luminol (5-amino-2,3-dihidro-1,4-phtal-azinedione) into a silica matrix in [374]. The dye is used in chemiluminescent analysis of biological objects [27], but poor solubility in water with neutral pH values hinders its wide application. To solubilize luminol, micelles were prepared from a biocompatible alkyl polyglycoside, the polar region of which contained glucose residues [374]. The surfactant served the dual function of solubilizing the dye and catalyzing the biomimetic silicification of alkyl polyglycoside micelles. Luminol not only held its properties after immobilization, but the intensity of its fluorescence increased more than 10 times.

Low molecular weight dyes are readily washed out of a silica matrix in an external solution. An approach in which dyes are covalently attached to polymers has become widespread. It was applied in [375]. Acid–base dyes xylenol orange and methyl red were attached to chitosan and neutral red, to carboxymethylcellulose. Polysaccharides are well entrapped in SiO_2_, and, in addition, they catalyze sol–gel synthesis and regulate the structure of the silica matrix (Section 7.3 and Section 7.5.2). The synthesized hybrid materials turned out to be optically transparent (Figure 16a), which made it possible to use UV–vis spectroscopy to study conjugated dyes and their spectral properties depending on the pH of contacting aqueous solutions. The best results were obtained with xylenol orange. Conjugation did not lead to a noticeable change in its spectra. This is explained by the fact that covalent addition was carried out at the carboxyl groups of the dye, which are not directly connected to the chromophore center. The functional groups of methyl red and neutral red used for conjugation are auxochromic, affecting the spectral characteristics of the dyes. In the case of xylenol orange, a material was produced that could find application as a pH sensor.

Tannic acid, the entrapment of which provides intensely colored silica (Figure 15b,c), can also find application as a pH sensor since its spectral characteristics change significantly when the acidity of the medium is varied. Complexes with metal ions are especially suitable for this purpose [376,377,378]. Tannic acid has broad potential for various functionalization of bionanocomposites, in the pores of which it is left after biomimetic synthesis. In particular, nanoparticles of silver and gold (Figure 15d) were synthesized, as well as nano-sized titanium dioxide in [356,357]. They were in a silica matrix and on the surface, demonstrating high catalytic and photocatalytic activities.

## 8. Conclusions

A review of biomimetic approaches to silicification of biopolymers and living cells has revealed their fairly wide variety. They have been proposed mainly in the last twenty years since the field began to develop relatively recently. Great interest is due to the new prospects that have opened up.

Living systems functioning at ambient conditions, in aquatic environments, and in the absence of toxic substances are of great interest for the development of highly efficient green technologies and materials with unique properties and functionality. However, incorporating biomaterial into a functionally suitable matrix faces enormous difficulties. At the same time, although methods for isolating polysaccharides, proteins, enzymes, and other biological substances are well developed, the problem of their inclusion in matrices with complete preservation of activity has not been fully solved. As shown in the review, sol–gel technology is one of the leading approaches. Its use is supported by the widespread occurrence of similar processes in living nature, the main example of which is diatoms that use silicic acid as a building material and have created highly efficient biosilicification processes. Traditional sol–gel technology is fundamentally different from them. Processes and precursors are poorly compatible with biomaterials. Therefore, the main efforts are aimed at improving and bringing them closer to the processes of living cells.

The analysis made in the review showed that great progress has been made in the development of biomimetic methods. Among the methods, two-stage synthesis should be highlighted (Section 6.1). It has been widely used for the immobilization of a great diversity of biomaterials in many publications. The method makes it possible to reduce or even completely eliminate the negative effects of TEOS or TMOS, which are the main precursors in sol–gel chemistry. However, the two-stage scheme (Figure 6) is labor-intensive and costly. This is a major drawback that limits its application in industry.

Precursors proposed for biomimetic immobilization of biomaterials to date are summarized in Table 1. A comparison of their advantages and disadvantages allows us to highlight THEOS. It has more of the former than the latter. Its synthesis, which consists of exchanging alcohol residues for ethylene glycol in TEOS and TMOS, is characterized by good reproducibility and yield approaching 100%. Silane is a liquid, which makes it easy to use. Sol–gel processes occur in one stage at a high rate at physiological pH without the addition of catalysts and organic solvents. No heating is required. Hydrolysis proceeds with the release of ethylene glycol, which does not lead to protein denaturation and polysaccharide precipitation. A variety of applications are shown in the review. The main disadvantage is poor compatibility with living cells due to the presence of ethylene glycol, to which they are very sensitive. This is a limiting factor for its widespread applications.

## 9. Future Directions

According to the author, who has wide experience in the development of biomimetic sol–gel methods, the most promising for biomimetic syntheses is the use of THEOS. It is characterized by both good compatibility with biomaterials and a simple one-step process under conditions most favorable for the immobilized proteins and polysaccharides. Research with it should be continued, which will lead to the development of new biocompatible materials with new functionality for biosensing, biotechnology, cosmetics, and biomedicine. At the same time, as shown in the review using a number of examples, there are good prospects for the development of materials for modern technology—optical, photonic, catalytic, and others.

An urgent problem that still remains practically unsolved is cell immobilization. The proposed approaches are far from optimal. Cells, with a few exceptions, are exposed to great stress during sol–gel synthesis, which leads to their short life span or even death during the process. New radical solutions are needed, which are being explored but are still at an early stage of development.

## Figures and Tables

**Table 1:** Silica precursors used for biomimetic silicification.

Silane	Synthesis	State	Sol–Gel Synthesis	Disadvantages	Biomimetic Potential	Refs.
TMOS/TEOS *	Commercial	Liquid	Acid/alkali and organic solvent addition, heating, two stages	Silane hydrophobic, protein denaturation, polysaccharide precipitation, cell lysis	Very poor	[7,9,20,22,49,75,76,77,78,79,80,81]
TGOS **	Transesterification, large excess of glycerol, catalyst	Solid, mixture of different isomers	Glycerol excess, two stages, catalyst	High viscosity, slow sol–gel processes	Good	[7,82,264,265,276,277]
Diglycerylsilane	Transesterification	Solid, individual substance	Two stages, catalyst	High viscosity, slow sol–gel processes	Good	[76,94,260,267,278]
THEOS ***	Transesterification	Liquid	One stage, neutral pH	Minimal	Good	[52,276,291,292,296,302,303]
Spirosiloxane ****	Transesterification, solution in benzene, catalyst or Esterification of silica, catalyst	Solid	Two stages, catalyst	--	Not studied	[293,295,297,298,301]
Polyethylene glycol-containing silane	Transesterification, PEG400	Liquid	99 wt.% silane, 80 °C or catalyst	High viscosity, slow sol–gel processes, polysaccharide precipitation	Poor	[275,280,281]
Sugar-containing silane *****	Sugars covalently attached to (aminopropyl)triethoxysilane	Solid	10% additive to diglycerylsilane or TEOS, two stages	High price, disadvantages of diglycerylsilane or TEOS	Good	[65,224,272,282]

* Tetramethoxysilane and tetraethoxysilane. ** Tetraglycerol orthosilicate. *** Tetrakis(2-hydroxyethyl)silane. **** Bis(ethane-1,2-diyldioxy)silane or chelate. ***** Gluconamidyltriethoxysilane and maltonamidyltriethoxysilane.

## Data Availability

Not applicable.

## References

[1] Greenwood N.N., Earnshaw A. (1998). Chemistry of the Elements.

[2] Tret’yakov Y.D., Martinenko L.I., Grigor’ev A.N., Zivadze A.Y. (2001). Inorganic Chemistry. Chemistry of Elements. Textbook.

[3] Treguer P., Nelson D.M., Van Bennekom A.J., DeMaster D.J., Leynaert A., Queguiner B. (1995). The silica balance in the world ocean: A reestimate. Science.

[4] Lobus N.V., Kulikovskiy M.S. (2023). The co-evolution aspects of the biogeochemical role of phytoplankton in aquatic ecosystems: A review. Biology.

[5] Lei Q., Guo J., Kong F., Cao J., Wang L., Zhu W., Brinker C.J. (2021). Bioinspired cell silicification: From extracellular to intracellular. J. Am. Chem. Soc..

[6] Patwardhan S.V. (2011). Biomimetic and bioinspired silica: Recent developments and applications. Chem. Commun..

[7] Gill I., Ballesteros A. (2000). Bioencapsulation within synthetic polymers (Part 1): Sol-gel encapsulated biologicals. Trends Biotechnol..

[8] Wallace J.M., Rice J.K., Pietron J.J., Stroud R.M., Long J.W., Rolison D.R. (2003). Silica nanoarchitectures incorporating self-organized protein superstructures with gas-phase bioactivity. Nano Lett..

[9] Brennan J.D. (2007). Biofriendly sol-gel processing for the entrapment of soluble and membrane-bound proteins: Toward novel solid-phase assays for high-throughput screening. Acc. Chem. Res..

[10] Livage J., Coradin T., Klein L., Aparicio M., Jitianu A. (2018). Encapsulation of enzymes, antibodies, and bacteria. Handbook of Sol-Gel Science and Technology.

[11] Guisan J.M., Lopez-Gallego F., Bolivar J.M., Rocha-Martin J., Fernandez-Lorente G., Guisan J.M., Bolivar J.M., Lopez-Galego F., Rocha-Martin J. (2020). The science of enzyme immobilization. Immobilization of Enzymes and Cells. Methods and Protocols.

[12] Rastegari E., Hsiao Y.J., Lai W.Y., Lai Y.H., Yang T.C., Chen S.J., Huang P.I., Chiou S.H., Mou C.Y., Chien Y. (2021). An update on mesoporous silica nanoparticle Applications in nanomedicine. Pharmaceutics.

[13] Melo J.S., Tripathi A., Kumar J., Mishra A., Sandaka P., Bhainsa C., Tripathi A., Melo J.S. (2021). Immobilization: Then and now. Immobilization Strategies. Biomedical, Bioengineering and Environmental Applications.

[14] Guo J., Amini S., Lei Q., Ping Y., Agola J.O., Wang L., Zhou L., Cao J., Franco S., Noureddine A. (2022). Robust and long-term cellular protein and enzymatic activity preservation in biomineralized mammalian cells. ACS Nano.

[15] Asaduzzaman F., Salmon S. (2022). Enzyme immobilization: Polymer-solvent-enzyme compatibility. Mol. Syst. Des. Eng..

[16] Scala-Benuzzi M.L., Palacios S.V.P., Takara E.A., Fernandez-Baldo M.A., Kumar K., Dash S.K., Ray S., Parween S. (2023). Biomaterials and biopolymers for the development of biosensors. Biomaterials-Based Sensors. Recent Advances and Applications.

[17] Marinheiro D., Martel F., Ferreira B.J.M.L., Daniel-da-Silva A.L. (2023). Silica-based nanomaterials for diabetes mellitus treatment. Bioengineering.

[18] Zhou Y., Liu K., Zhang H. (2023). Biomimetic mineralization: From microscopic to macroscopic materials and their biomedical applications. ACS Appl. Biomater..

[19] Livage J. (1997). Sol-gel processes. Curr. Opin. Solid State Mater. Sci..

[20] Avnir D., Klein L.C., Levy D., Schubert U., Wojcik A.B., Rappoport Z., Apeloig W. (1998). Organo-silica sol-gel materials. The Chemistry of Organic Silicon Compounds.

[21] Bhatia R.B., Brinker C.J., Gupta A.K., Singh A.K. (2000). Aqueous sol-gel process for protein encapsulation. Chem. Mater..

[22] Livage J., Coradin T., Roux C. (2001). Encapsulation of biomolecules in silica gels. J. Phys.-Condens. Matter.

[23] Jin W., Brennan J.D. (2002). Properties and applications of proteins encapsulated within sol-gel derived materials. Anal. Chim. Acta.

[24] Rickus J.L., Dunn B., Zink J.I., Ligler F.S., Taitt C.A.R. (2002). Optically based sol-gel biosensor materials. Optical Biosensors: Present and Future.

[25] Wight A.P., Davis M.E. (2002). Design and preparation of organic-norganic hybrid catalysts. Chem. Rev..

[26] Pierre A.C. (2004). The sol-gel encupsulation of enzymes. Biocatal. Biotrans..

[27] Kandimalla V.B., Tripathi V.S., Ju H.X. (2006). Immobilization of biomolecules in sol-gels: Biological and analytical applications. Crit. Rev. Anal. Chem..

[28] Besanger T.R., Brennan J.D. (2006). Entrapment of membrane proteins in sol-gel derived silica. J. Sol-Gel Sci. Technol..

[29] Forsyth C., Patwardhan S.V., Zelisko P.M. (2014). Bioinspired silica for enzyme immobilisation: A comparison with traditional methods. Bio-Inspired Silicon-Based Materials.

[30] Owens G.J., Singh R.K., Foroutan F., Alqaysi M., Han C.M., Mahapatra C., Kim H.W., Knowles J.C. (2016). Sol-gel based materials for biomedical applications. Prog. Mater. Sci..

[31] Nassar E.J., Ciuffi K.J., Calefi P.S., Rocha L.A., De Faria E.H., Silva M.L.A., Luz P.P., Bandeira L.C., Cestari A., Fernandes C.N., Pignatello R. (2011). Biomaterials and sol-gel process: A methodology for the preparation of functional materials. Biomaterials Science and Engineering.

[32] Boury B., Plumejeau S. (2015). Metal oxides and polysaccharides: An efficient hybrid association for materials chemistry. Green Chem..

[33] Shchipunov Y., Postnova I. (2018). Cellulose mineralization as a route for novel functional materials. Adv. Funct. Mater..

[34] An B., Wang Y., Huang Y., Wang X., Liu Y., Xun D., Church G.M., Dai Z., Yi X., Tang T.C. (2023). Engineered living materials for sustainability. Chem. Rev..

[35] Shchipunov Y. (2012). Bionanocomposites: Green sustainable materials for the near future. Pure Appl. Chem..

[36] Iler R.K. (1979). The Chemistry of Silica: Solubility, Polymerization, Colloid and Surfaces Properties, and Biochemistry.

[37] Brinker C.J., Scherer G.W. (1990). Sol-Gel Science. The Physics and Chemistry of Sol-Gel Processing.

[38] Pierre A.C. (1998). Introduction to Sol-Gel Processing.

[39] Sanchez C., Lebeau B., Ribot F. (2000). Molecular design of sol-gel derived hybrid organic-inorganic nanocomposites. J. Mater.Chem..

[40] Caruso R.A., Antonietti M. (2001). Sol-gel nanocoating: An approach to the preparation of structured materials. Chem. Mater..

[41] Van Bommel K.J.C., Friggeri A., Shinkai S. (2003). Organic templates for the generation of inorganic materials. Angew. Chem. Int. Ed..

[42] Coradin T., Boissiere M., Livage J. (2006). Sol-gel chemistry in medicinal science. Curr. Med. Chem..

[43] Ciriminna R., Fidalgo A., Pandarus V., Beland F., Ilharco L.M., Pagliaro M. (2013). The sol-gel route to advanced silica-based materials and recent applications. Chem. Rev..

[44] Homburg S.V., Patel A.V. (2022). Silica hydrogels as entrapment material for microalgae. Polymers.

[45] Da Cruz Schneid A., Albuquerque L.J.C., Mondo G.B., Ceolin M., Picco A.S., Cardoso M.B. (2022). Colloidal stability and degradability of silica nanoparticles in biological fluids: A review. J. Sol-Gel Sci. Technol..

[46] Lee J.E., Lee N., Kim T., Kim J., Hyeon T. (2011). Multifunctional mesoporous silica nanocomposite nanoparticles for theranostic applications. Acc. Chem. Res..

[47] Chircov C., Spoiala A., Paun C., Craciun L., Ficai D., Ficai A., Andronescu E., Turculet L.C. (2020). Mesoporous silica platforms with potential applications in release and adsorption of active agents. Molecules.

[48] Hench L.L. (1998). Sol-Gel Silica. Properties, Processing and Technology Transfer.

[49] Ellerby L.M., Nishida C.R., Nishida F., Yamanaka S.A., Dunn B., Valentine J.S., Zink J.I. (1992). Encapsulation of proteins in transparent porous silicate-glasses prepared by the sol-gel method. Science.

[50] Shchipunov Y.A., Karpenko T.Y., Krekoten A.V. (2005). Hybrid organic-inorganic nanocomposites fabricated with a novel biocompatible precursor using sol-gel processing. Compos. Interfaces.

[51] Sui X.H., Cruz-Aguado J.A., Chen Y., Zhang Z., Brook M.A., Brennan J.D. (2005). Properties of human serum albumin entrapped in sol-gel-derived silica bearing covalently tethered sugars. Chem. Mater..

[52] Shchipunov Y.A., Ruiz-Hitzky E., Ariga K., Lvov Y. (2008). Entrapment of biopolymers into sol-gel-derived silica nanocomposites. Bio-Inorganic Hybrid Nanomaterials.

[53] Wanka R., Koc J., Clarke J., Hunsucker K.Z., Swain G.W., Aldred N., Finlay J.A., Clare A.S., Rosenhahn A. (2020). Sol-gel-based hybrid materials as antifouling and fouling-release coatings for marine applications. ACS Appl. Mater. Interfaces.

[54] Kroger N., Lehmann G., Rachel R., Sumper M. (1997). Characterization of a 200-kDa diatom protein that is specifically associated with a silica-based substructure of the cell wall. Eur. J. Biohem..

[55] Shimizu K., Cha J., Stucky G.D., Morse D.E. (1998). Silicatein alpha: Cathepsin L-like protein in sponge biosilica. Proc. Natl. Acad. Sci. USA.

[56] Cha J.N., Chimizu K., Zhou Y., Christiansen S.C., Chmelka B.F., Stucky G.D., Morse D.E. (1999). Silicatein filaments and subunits from a marine sponge direct the polymerization of silica and silicones in vitro. Proc. Natl. Acad. Sci. USA.

[57] Kroger N., Deutzmann R., Sumper M. (1999). Polycationic peptides from diatom biosilica that direct silica nanosphere formation. Science.

[58] Kroger N., Deutzmann R., Bergsdorf C., Sumper M. (2000). Species-specific polyamines from diatoms control silica morphology. Proc. Natl. Acad. Sci. USA.

[59] Kroger N., Deutzmann R., Sumper M. (2001). Silica-precipitating peptides from diatoms. J. Biol. Chem..

[60] Maksimov A.I., Moshnikov V.A., Tairov Y.M., Shilova O.A. (2007). The Elements of Sol-Gel Technology of Nanocomposites.

[61] Lu A.H., Zhao D., Wan Y. (2010). Nanocasting. A Versatile Strategy for Creating Nanostructured Porous Materials.

[62] Esposito S. (2023). Sol–Gel Synthesis Strategies for Tailored Catalytic Materials.

[63] Alexander G.B. (1954). The polymerization of monosilicic acid. J. Am. Chem. Soc..

[64] Greenberg S.A. (1959). The chemistry of silicic acid. J. Chem. Educ..

[65] Lin T.Y., Wu C.H., Brennan J.D. (2007). Entrapment of horseradish peroxidase in sugar-modified silica monoliths: Toward the development of a biocatalytic sensor. Biosens. Bioelectron..

[66] Hui C.Y., Lau P.S., Li Y., Brennan J.D. (2019). Investigation of RNA structure-switching aptamers in tunable sol-gel-derived materials. J. Sol-Gel Sci. Technol..

[67] Pierre A.C., Pajonk G.M. (2002). Chemistry of aerogels and their applications. Chem. Rev..

[68] Schmidt H., Scholze H., Kaiser A. (1984). Principles of hydrolysis and condensation reaction of alkoxysilanes. J. Non-Cryst. Solids.

[69] Hench L.L., West J.K. (1990). The sol-gel process. Chem. Rev..

[70] Arkles B. (1997). Silicon esters. Kirk-Othmer Encyclopedia of Chemical Technology.

[71] Tripathi V.S., Kandimalla V.B., Ju H. (2006). Preparation of ormosil and its applications in the immobilizing biomolecules. Sens. Actuat. B Chem..

[72] Gutierrez-Climente R., Clavie M., Dumy P., Mehdi A., Subra G. (2021). Sol-gel process: The inorganic approach in protein imprinting. J. Mater. Chem. B.

[73] Ebelmen M. (1846). Sur les combinaisons des acides borique et silicique avec les éthers. Ann. Chim. Phys..

[74] Wu S.G., Ellerby L.M., Cohan J.S., Dunn B., Elsayed M.A., Valentine J.S., Zink J.I. (1993). Bacteriorhodopsin encapsulated in transparent sol-gel glass—A new biomaterial. Chem. Mater..

[75] Brook M.A., Chen Y., Guo K., Zhang Z., Brennan J.D. (2004). Sugar-modified silanes: Precursors for silica monoliths. J. Mater. Sci..

[76] Johnson G.R., Luckarift H.R., Minteer S.D. (2017). Enzyme stabilization via bio-templated silicification reactions. Enzyme Stabilization and Immobilization: Methods and Protocols.

[77] Zaremba C.M., Stucky G.D. (1996). Biosilicates and biomimetic silicate synthesis. Curr. Opin. Solid. State. Mat. Sci..

[78] Livage J., Coradin T., Roux C., Gomez-Romero P., Sanchez C. (2004). Bioactive sol-gel hybrids. Functional Hybrid Materials.

[79] Coradin T., Livage J. (2007). Aqueous silicates in biological sol-gel applications: New perspectives for old precursors. Acc. Chem. Res..

[80] Baccile N., Babonneau F., Thomas B., Coradin T. (2009). Introducing ecodesign in silica sol-gel materials. J. Mater. Chem..

[81] Povarova N.V., Markina N.M., Baranov M.S., Barinov N.A., Klinov D.V., Kozhemyako V.B., Lukyanov K.A. (2018). A water-soluble precursor for efficient silica polymerization by silicateins. Biochem. Biophys. Res. Comm..

[82] Bruno F., Gigli L., Ferraro G., Cavallo A., Michaelis V.K., Goobes G., Fratini E., Ravera E. (2022). Lysozyme is sterically trapped within the silica cage in bioinspired silica-lysozyme composites: A multi-technique understanding of elusive protein-material Interactions. Langmuir.

[83] Bruno F., Gigli L., Ravera E. (2023). Spin label study of the orientational preferences of lysozyme in a bioinspired silica composite. J. Compos. Sci..

[84] Lapanie S. (1978). Physico-Chemical Aspects of Protein Denaturation.

[85] Miller J.M., Dunn B., Valentine J.S., Zink J.I. (1996). Synthesis conditions for encapsulating cytochrome c and catalase in SiO_2_ sol-gel materials. J. Non-Cryst. Solids.

[86] Dunn B., Miller J.M., Dave B.C., Valentine J.S., Zink J.I. (1998). Strategies for encapsulating biomolecules in sol-gel matrix. Acta Mater..

[87] Ferrer M.L., Del Monte F., Mateo C.R., Gomez J., Levy D. (2003). Denaturation and leaching study of horseradish peroxidase encapsulated in sol-gel matrices. J. Sol-Gel Sci. Technol..

[88] Coiffier A., Coradin T., Roux C., Bouvet O.M.M., Livage J. (2001). Sol-gel encapsulation of bacteria: A comparison between alkoxide and aqueous routes. J. Mater. Chem..

[89] Stelzer G.I., Klug E.D., Davidson R.L. (1980). Carboxymethylcellulose. Handbook of Water-Soluble Gums and Resins.

[90] Roberts G.A.F. (1992). Chitin Chemistry.

[91] Ahmed S. (2019). Alginates. Applications in the Biomedical and Food Industries.

[92] Belitz H.D., Grosch W., Schieberle P. (2009). Food Chemistry.

[93] Brook M.A., Brennan J.D., Cheng Y. (2003). Polyol-Modified Silanes as Precursors for Silica. Canada Patent.

[94] Flora K.K., Brennan J.D. (2001). Effect of matrix aging on the behavior of human serum albumin entrapped in a tetraethyl orthosilicate-derived glass. Chem. Mater..

[95] Sola-Rabada A., Rinck J., Belton D.J., Powell A.K., Perry C.C. (2016). Isolation of a wide range of minerals from a thermally treated plant: Equisetum arvense, a Mare’s tale. J. Biol. Inorg. Chem..

[96] Davy H. (1815). The Elements of Agricultural Chemistry.

[97] Currie H.A., Perry C.C. (2007). Silica in plants: Biological, biochemical and chemical studies. Ann. Bot..

[98] Guerriero G., Hausman J.-F., Legay S. (2016). Silicon and the plant extracellular matrix. Front. Plant Sci..

[99] Sommer A.L. (1926). Studies Concerning Essential Nature of Aluminum and Silicon for Plant Growth.

[100] Raleigh G.J. (1939). Evidence for the essentiality of silicon fro growth of the beet plant. Plant Physiol..

[101] Lewin J., Reimann B.E.F. (1969). Silicon and plant growth. Annu. Rev. Plant Physiol..

[102] Chen C.-H., Lewin J. (1969). Silicon as a nutrient element for *Equisetum arvense*. Can. J. Bot..

[103] Ma J.F., Bauerlein E. (2007). Uptake of silicon in different plant species. Handbook of Biomineralization.

[104] Ma J., Cai H.M., He C.W., Zhang W.J., Wang L.J. (2015). A hemicellulose-bound form of silicon inhibits cadmium ion uptake in rice (*Oryza sativa*) cells. New Phytol..

[105] Ma J., Zhang X.Q., Zhang W.J., Wang L.J. (2016). Multifunctionality of silicified nanoshells at cell interfaces of *Oryza sativa*. ACS Sustain. Chem. Eng..

[106] Nelson D.M., Treguer P., Brzezinski M.A., Leynaert A., Queguiner B. (1995). Production and dissolution of biogenic silica in the ocean: Revised global estimates, comparison with regional data and relationship to biogenic sedimentation. Glob. Biogeochem. Cycles.

[107] Frausto da Silva J.J.R., Williams R.J.P. (2001). The Biological Chemistry of the Elements. The Inorganic Chemistry of Life.

[108] Brandstadt K.F. (2005). Inspired by nature: An exploration of biocatalyzed siloxane bond formation and cleavage. Curr. Opin. Biotechnol..

[109] Schroder H.C., Wang X.H., Tremel W., Ushijima H., Muller W.E.G. (2008). Biofabrication of biosilica-glass by living organisms. Nat. Prod. Rep..

[110] Nudelman F., Sommerdijk N.A.J.M. (2012). Biomineralization as an Inspiration for materials chemistry. Angew. Chem. Int. Ed..

[111] Sullivan C.W., Volcani B.E., Simpson T.L., Volcani B.E. (1981). Silieon in the cellular metabolism of diatoms. Silicon and Siliceous Structures in Biological Systems.

[112] Sumper M., Brunner E. (2006). Learning from diatoms: Nature’s tools for the production of nanostructured silica. Adv. Funct. Mater..

[113] Khan M.J., Rai A., Ahirwar A., Sirotiya V., Mourya M., Mishra S., Schoefs B., Marchand J., Bhatia S.K., Varjani S. (2021). Diatom microalgae as smart nanocontainers for biosensing wastewater pollutants: Recent trends and innovations. Bioengineered.

[114] Rogato A., De Tommasi E. (2020). Physical, chemical, and genetic techniques for diatom frustule modification: Applications in nanotechnology. Appl. Sci..

[115] Kroger N., Lorenz S., Bruner E., Sumper M. (2002). Self-assembly of highly phosphorylated silaffins and their function in biosilica morphogenesis. Science.

[116] Haeckel E.H. (1904). Kunstformen Der Natur.

[117] Lechner C., Becker C. (2015). Silaffins in silica biomineralization and biomimetic silica precipitation. Mar. Drugs.

[118] Morse D.E. (1999). Silicon biotechnology: Harnessing biological silica production to construct new materials. Trends Biotechnol..

[119] Aizenberg J., Sundar V.C., Yablon A.D., Weaver J.C., Chen G. (2004). Biological glass fibers: Correlation between optical and structural properties. Proc. Natl. Acad. Sci. USA.

[120] Aizenberg J., Weaver J.C., Thanawala M.S., Sundar V.C., Morse D.E., Fratzl P. (2005). Skeleton of *Euplectella* sp.: Structural hierarchy from the nanoscale to the macroscale. Science.

[121] Brutchey R.L., Morse D.E. (2008). Silicatein and the translation of its molecular mechanism of biosilicification into low temperature nanomaterial synthesis. Chem. Rev..

[122] Povarova N.V., Barinov N.A., Baranov M.S., Markina N.M., Varizhuk A.M., Pozmogova G.E., Klimov D.V., Kozhemyako V.B., Lukyanov K.A. (2018). Efficient silica synthesis from tetra(glycerol)orthosilicate with cathepsin- and silicatein-like proteins. Sci. Rep..

[123] Cha J.N., Stucky G.D., Morse D.E., Deming T.J. (2000). Biomimetic synthesis of ordered silica strutures mediated by block copolypeptides. Nature.

[124] Patwardhan S.V., Mukherjee N., Clarson S.J. (2001). The use of poly-L-lysine to form novel silica morphologies and the role of polypeptides in biosilicification. J. Inorg. Organomet. Polym..

[125] Patwardhan S.V., Clarson S.J. (2002). Silicification and biosilicification—Part 1. Formation of silica structures utilizing a cationically charged synthetic polymer at neutral pH and under ambient conditions. Polym. Bull..

[126] Patwardhan S.V., Clarson S.J. (2002). Silicification and biosilicification—Part 4. Effect of template size on the formation of silica. J. Inorg. Organomet. Polym..

[127] Patwardhan S.V., Clarson S.J. (2003). Silicification and biosilicification—Part 7: Poly-L-arginine mediated bioinspired synthesis of silica. J. Inorg. Organomet. Polym..

[128] Patwardhan S.V., Clarson S.J. (2003). Silicification and biosilicification: Part 5—An investigation of the silica structures formed at weakly acidic pH and neutral pH as facilitated by cationically charged macromolecules. Mater. Sci. Eng. C.

[129] Patwardhan S.V., Clarson S.J. (2003). Silicification and biosilicification—Part 6. Poly- L-histidine mediated synthesis of silica at neutral pH. J. Inorg. Organomet. Polym..

[130] Patwardhan S.V., Mukherjee N., Steinitz-Kannan M., Clarson S.J. (2003). Bioinspired synthesis of new silica structures. Chem. Commun..

[131] Coradin T., Livage J. (2001). Effect of some amino acids and peptides on silicic acid polymerization. Colloid Surf. B.

[132] Coradin T., Durupthy O., Livage J. (2002). Interactions of amino-containing peptides with sodium silicate and colloidal silica: A biomimetic approach of silicification. Langmuir.

[133] Coradin T., Roux C., Livage J. (2002). Biomimetic self-activated formation of multi-scale porous silica in the presence of arginine-based surfactants. J. Mater. Chem..

[134] Naik R.R., Whitlock P.W., Rodriguez F., Brott L.L., Glawe D.D., Clarson S.J., Stone M.O. (2003). Controlled formation of biosilica structures in vitro. Chem. Commun..

[135] Mizutani T., Nagase H., Ogoshi H. (1998). Silicic acid polymerization catalyzed by amines and polyamines. Chem. Lett..

[136] Mizutani T., Nagase H., Fujiwara N., Ogoshi H. (1998). Silicic acid polymerization catalyzed by amines and polyamines. Bull. Chem. Soc. Jpn..

[137] Coffman E.A., Melechko A.V., Allison D.P., Simpson M.L., Doktycz M.J. (2004). Surface patterning of silica nanostructures using bio-inspired templates and directed synthesis. Langmuir.

[138] Noll F., Sumper M., Hampp N. (2002). Nanostructure of diatom silica surfaces and of biomimetic analogues. Nano Lett..

[139] Vrieling E.G., Beelen T.P.M., van Santen R.A., Gieskes W.W.C. (2002). Mesophases of (bio)polymer-silica particles inspire a model for silica biomineralization in diatoms. Angew. Chem. Int. Ed..

[140] Li C., Kaplan D.L. (2003). Biomimetic composites via molecular scale self-assembly and biomineralization. Curr. Opin. Solid State Mater. Sci..

[141] Sun Q., Vrieling E.G., van Santen R.A., Sommerdijk N.A.J.M. (2004). Bioinspired synthesis of mesoporous silicas. Curr. Opin. Solid State Mater. Sci..

[142] Patwardhan S.V., Shiba K., Raab C., Husing N., Clarson S.J. (2005). Protein-mediated bioinspired mineralization. ACS Symp. Ser..

[143] Schroder H.C., Brandt D., Schlossmacher U., Wang X.H., Tahir M.N., Tremel W., Belikov S.I., Muller W.E.G. (2007). Enzymatic production of biosilica glass using enzymes from sponges: Basic aspects and application in nanobiotechnology (material sciences and medicine). Naturwissenschaften.

[144] Dickerson M.B., Sandhage K.H., Naik R.R. (2008). Protein- and peptide-directed syntheses of inorganic materials. Chem. Rev..

[145] Muller W.E.G., Wang X.H., Cui F.Z., Jochum K.P., Tremel W., Bill J., Schroder H.C., Natalio F., Schlossmacher U., Wiens M. (2009). Sponge spicules as blueprints for the biofabrication of inorganic-organic composites and biomaterials. Appl. Microbiol. Biotechnol..

[146] Cannavale A., Fiorito F., Manca M., Tortorici G., Cingolani R., Gigli G. (2010). Multifunctional bioinspired sol-gel coatings for architectural glasses. Build. Environ..

[147] Maddala S.P., Liao W.C., Joosten R.R.M., Soleimani M., Tuinier R., Friedrich H., van Benthem R.A.T.M. (2021). Chain length of bioinspired polyamines affects size and condensation of monodisperse silica particles. Commun. Chem..

[148] Wallace A.K., Chanut N., Voigt C.A. (2020). Silica nanostructures produced using diatom peptides with designed post-translational modifications. Adv. Funct. Mater..

[149] Sudheendra L., Raju A.R. (2002). Peptide-induced formation of silica from tetraethylorthosilicate at near-neutral pH. Mater. Res. Bull..

[150] Van Bommel K.J.C., Jung J.H., Shinkai S. (2001). Poly(L-lysine) aggregates as templates for the formation of hollow silica spheres. Adv. Mater..

[151] Reetz M.T., Zonta A., Simpelkamp J. (1995). Efficient heterogeneous biocatalysts by entrapment of lipases in hydrophobic sol-gel materials. Angew. Chem. Int. Ed..

[152] Kuncova G., Guglielmi M., Dubina P., Safar B. (1995). Lipase immobilized by sol-gel technique in layers. Collect. Czech. Chem. Commun..

[153] Reetz M.T., Zonta A., Simpelkamp J., Rufinska A., Tesche B. (1996). Characterization of hydrophobic sol-gel materials containing entrapped lipases. J. Sol-Gel Sci. Technol..

[154] Reetz M.T., Zonta A., Simpelkamp J. (1996). Efficient immobilization of lipases by entrapment in hydrophobic sol-gel materials. Biotechnol. Bioeng..

[155] Buisson P., El Rassy H., Maury S., Pierre A.C. (2003). Biocatalytic gelation of silica in the presence of a lipase. J. Sol-Gel Sci. Technol..

[156] Pierre A.C., Buisson P. (2006). Use of a lipase to synthesize silica gels in a hydrophobic organic solvent. J. Sol-Gel Sci. Technol..

[157] Kawakami K., Yoshida S. (1994). Entrapment of lipase in silica glass by the sol-gel method and its esterification activity in organic media. Biotechnol. Technol..

[158] Embleton J.K., Pouton C.W. (1997). Structure and function of gastro-intestinal lipases. Adv. Drug Deliv. Rev..

[159] Voet D., Voet J.G., Pratt C.W. (2008). Fundamentals of Biochemistry. Life at the Molecular Level.

[160] Reetz M.T. (1997). Entrapment of biocatalysts in hydrophobic sol-gel materials for use in organic chemistry. Adv. Mater..

[161] Maury S., Buisson P., Pierre A.C. (2002). Catalytic activity of *Burkholderia* (Pseudomonas) *cepacia* encapsulated in silica aerogels in esterification and hydrolysis as a function of the gel and solvent hydrophobicities. J. Mol. Catal. B Enzym..

[162] Nishino H., Mori T., Okahata Y. (2002). Enzymatic silicone oligomerization catalyzed by a lipid-coated lipase. Chem. Commun..

[163] Bassindale A.R., Brandstadt F.K., Lane T.H., Taylor P.G. (2003). Enzyme-catalysed siloxane bond formation. J. Inorg. Biochem..

[164] Perry C.C., Keeling-Tucker T. (2000). Biosilicification: The role of the organic matrix in structure control. J. Biol. Inorg. Chem..

[165] Mann S. (2002). Biomineralization: Principles and Concepts in Bioinorganic Materials Chemistry.

[166] Hildebrand M. (2008). Diatoms, biomineralization processes, and genomics. Chem. Rev..

[167] Allison D.P., Dufrene Y.F., Doktycz M.J., Hildebrand M. (2008). Biomineralization at the nanoscale: Learning from diatoms. Methods Cell Biol..

[168] Brunner E., Groger C., Lutz K., Richthammer P., Spinde K., Sumper M. (2009). Analytical studies of silica biomineralization: Towards an understanding of silica processing by diatoms. Appl. Microbiol. Biotechnol..

[169] Clark S.G., Holt P.F., Went C.W. (1957). The interaction of silicic acid with insulin, albumin and nylon monolayers. Trans. Faraday Soc..

[170] Volkani B.E., Simpson T.L., Volkani B.E. (1981). Cell wall formation in diatoms: Morphogenesis and biochemistry. Silicon and Siliceous Structures in Biological Systems.

[171] Gordon R., Drum R.W. (1994). The chemical basis of diatom morphogenesis. Int. Rev. Cytol..

[172] Graham T. (1864). XXXV.On the properties of silicic acid and other analogous colloidal substances. J. Chem. Soc..

[173] Willstatter R., Kraut H., Lobinger K. (1928). UÜber die einfachsten Kieselsauren; mit Bemerkungen uber Aluminiumhydroxyde (XI. Mitteilung uber Hydrate und Hydrogele). Ber. Dtsch. Chem. Ges..

[174] Schulman J.H., Rideal E.K. (1937). Molecular interaction in monolayers. II The action of haemolytic and agglutinating agents on lipo-protein monolayers. Proc. R. Soc. Lond. B.

[175] Iler R.K. (1952). Association between polysilicic acid and polar organic compounds. J. Phys. Chem..

[176] Dickey F.H. (1955). Specific adsorption. J. Phys. Chem..

[177] Johnson P., Whateley T.L. (1971). On the use of polymerizing silica gel systems for the immobilization of trypsin. J. Colloid Interface Sci..

[178] Venton D.L., Cheesman K.L., Chatterton R.T., Anderson T.L. (1984). Entrapment of a highly specific antiprogesterone antiserum using polysiloxane copolymers. Biochim. Biophys. Acta.

[179] Glad M., Norrlow O., Sellergren B., Siegbahn N., Mosbach K. (1985). Use of silane monomers for molecular imprinting and enzyme entrapment in polysiloxane-coated porous silica. J. Chromatogr. A.

[180] Carturan G., Campostrini R., Diré S., Scardi V., De Alteriis E. (1989). Inorganic gels for immobilization of biocatalysts: Inclusion of invertase-active whole cells of yeast (*Saccharomyces cerevisiae*) into thin layers of SiO_2_ gel deposited on glass sheets. J. Mol. Catal. Lett..

[181] Braun S., Shtelzer S., Rappoport S., Avnir D., Ottolenghi M. (1992). Biocatalysis by sol-gel entrapped enzymes. J. Non-Cryst. Solids..

[182] Inama L., Diré S., Carturan G., Cavazza A. (1993). Entrapment of viable microorganisms by SiO_2_ sol-gel layers on glass surfaces: Trapping, catalytic performance and immobilization durability of *Saccharomyces cerevisiae*. J. Biotechnol..

[183] Braun S., Rappoport S., Zusman R., Avnir D., Ottolenghi M. (1990). Biochemically active sol-gel glasses: The trapping of enzymes. Mater. Lett..

[184] Yamanaka S.A., Nishida F., Ellerby L.M., Nishida C.R., Dunn B., Valentine J.S., Zink J.I. (1992). Enzymatic-activity of glucose-oxidase encapsulated in transparent glass by the sol-gel method. Chem. Mater..

[185] Audebert P., Sanches C. (1994). Modified electrodes from hydrophobic alkoxide silica gels—Insertion of electroactive compounds as glucose oxidase. J. Sol-Gel Sci. Technol..

[186] Collino R., Therasse J., Binder P., Chaput F., Boilot J.P. (1994). Thin films of functionalized amorphous silica for immunosensors application. J. Sol-Gel Sci. Technol..

[187] Heichal-Segal O., Rappoport S., Braun S. (1995). Immobilization in alginate-silicate sol-gel matrix protects beta-glucosidase against thermal and chemical denaturation. Nat. Biotechnol..

[188] Pope E.J.A. (1995). Gel encapsulated microorgansims: Saccharomyces cerevisiae-silica gel biocomposites. J. Sol-Gel Sci. Technol..

[189] Zuhlke J., Knopp D., Niessner R. (1995). Sol-gel glass as a new support matrix in immunoaffinity chromatography. Fresenius. J. Anal. Chem..

[190] Livage J., Roux C., Da Costa J.M., Desportes I., Quinson J.F. (1996). lmmunoassays in sol-gel matrices. J. Sol-Gel Sci. Technol..

[191] Li J., Tan S.N., Ge H.L. (1996). Silica sol-gel immobilized amperometric biosensor for hydrogen peroxide. Anal. Chim. Acta.

[192] Yamanaka S.A., Charych D.H., Loy D.A., Sasaki D.Y. (1997). Solid phase immobilization of optically responsive liposomes in sol-gel materials for chemical and biological sensing. Langmuir.

[193] Altstein M., Segev G., Aharonson N., Ben-Aziz O., Turniansky A., Avnir D. (1998). Sol-gel-entrapped cholinesterases: A microtiter plate method for monitoring anti-cholinesterase compounds. J. Agric. Food. Chem..

[194] Chen Q., Kenausis G.L., Heller A. (1998). Stability of oxidases immobilized in silica gels. J. Am. Chem. Soc..

[195] Schuleit M., Luisi P.L. (2001). Enzyme immobilization in silica-hardened organogels. Biotechnol. Bioeng..

[196] Premkumar J.R., Lev O., Rosen R., Belkin S. (2001). Encapsulation of Luminous Recombinant *E. coli* in Sol-Gel Silicate Films. Adv. Mater..

[197] Coradin T., Mercey E., Lisnard L., Livage J. (2001). Design of silica-coated microcapsules for bioencapsulation. Chem. Commun..

[198] Dunn B., Zink J.I. (1991). Optical-properties of sol-gel glasses doped with organic molecules. J. Mater. Chem..

[199] Weetall H.H. (1993). Preparation of immobilized proteins covalently coupled through silane coupling agents to inorganic supports. Appl. Biochem. Biotechnol..

[200] Avnir D., Braun S., Lev O., Ottolenghi M. (1994). Enzymes and other proteins entrapped in sol-gel materials. Chem. Mater..

[201] Dave B.C., Dunn B., Valentine J.S., Zink J.I. (1994). Sol-gel encapsulation methods for biosensors. Anal. Chem..

[202] Dave B.C., Miller J.M., Dunn B., Valentine J.S., Zink J.I. (1997). Encapsulation of proteins in bulk and thin film sol-gel matrices. J. Sol-Gel Sci. Technol..

[203] Gill I. (2001). Bio-doped nanocomposite polymers: Sol-gel bioencapsulates. Chem. Mater..

[204] Kim J., Grate J.W., Wang P. (2006). Nanostructures for enzyme stabilization. Chem. Eng. Sci..

[205] Sakai-Kato K., Ishikura K. (2009). Integration of biomolecules into analytical systems by means of silica sol-gel technology. Anal. Sci..

[206] Pierre A.C. (2019). From random glass networks to random silica gel networks and their use as host for biocatalytic applications. J. Sol-Gel Sci. Technol..

[207] Lei Q., Guo J., Noureddine A., Wang A., Wuttke S., Brinker C.J., Zhu W. (2020). Sol-gel-based advanced porous silica materials for biomedical applications. Adv. Funct. Mater..

[208] Jackson E., Correa S., Betancor L., Guisan J.M., Bolivar J.M., Lopez-Gallego F., Rocha-Martin J. (2020). In situ immobilization of enzymes in biomimetic silica. Immobilization of Enzymes and Cells: Methods and Protocols.

[209] Ortiz C., Jackson E., Betancor L. (2022). Immobilization and stabilization of enzymes using biomimetic silicification reactions. J. Sol-Gel Sci. Technol..

[210] Livage J. (1996). Bioactivity in sol-gel glasses. C. R. Acad. Sci. Ser. II.

[211] Gill I., Ballesteros A. (2000). Bioencapsulation within synthetic polymers (Part 2): Non-sol-gel protein-polymer biocomposites. Trends Biotechnol..

[212] Nassif N., Bouvet O., Rager M.N., Roux C., Coradin T., Livage J. (2002). Living bacteria in silica gels. Nat. Mater..

[213] Carturan G., Dal Toso R., Boninsegna S., Dal Monte R. (2004). Encapsulation of functional cells by sol-gel silica: Actual progress and perspectives for cell therapy. J. Mater. Chem..

[214] Kim J., Jia H.F., Wang P. (2006). Challenges in biocatalysis for enzyme-based biofuel cells. Biotechnol. Adv..

[215] Sahney R., Anand S., Puri B.K., Srivastava A.K. (2006). A comparative study of immobilization techniques for urease on glass-pH-electrode and its application in urea detection in blood serum. Anal. Chim. Acta.

[216] Gupta R., Chaudhury N.K. (2007). Entrapment of biomolecules in sol-gel matrix for applications in biosensors: Problems and future prospects. Biosens. Bioelectron..

[217] Frenkel-Mullerad H., Ben-Knaz R., Avnir D. (2014). Preserving the activity of enzymes under harsh oxidizing conditions: Sol-gel entrapped alkaline phosphatase exposed to bromine. J. Sol-Gel Sci. Technol..

[218] Luckarift H.R., Spain J.C., Naik R.R., Stone M.O. (2004). Enzyme immobilization in a biomimetic silica support. Nat. Biotechnol..

[219] Zhou L., Lei Q., Guo J., Gao Y., Shi J., Yu H., Yin W., Cao J., Xiao B., Andreo J. (2022). Long-term whole blood DNA preservation by cost-efficient cryosilicification. Nat. Commun..

[220] Besanger T.R., Chen Y., Deisingh A.K., Hodgson R., Jin W., Mayer S., Brook M.A., Brennan J.D. (2003). Screening of inhibitors using enzymes entrapped in sol-gel-derived materials. Anal. Chem..

[221] Ciesla U., Schuth F. (1999). Ordered mesoporous materials. Micropor. Mesopor. Mater..

[222] Harrell T.M., Hosticka B., Power M.E., Cemke L., Hull R., Norris P.M. (2004). Selective deposition of biocompatible sol-gel materials. J. Sol-Gel Sci. Technol..

[223] Ferrer M.L., Del Monte F., Levy D. (2002). A novel and simple alcohol-free sol-gel route for encapsulation of labile proteins. Chem. Mater..

[224] Ferrer M.L., Garcia-Carvajal Z.Y., Yuste L., Rojo F., Del Monte F. (2006). Bacteria viability in sol-gel materials revisited: Cryo-SEM as a suuitable tool to study the structural integrity of encapsulated bacteria. Chem. Mater..

[225] Jaroch D., McLamore E., Zhang W., Shi J., Garland J., Banks M.K., Porterfield D.M., Rickus J.L. (2011). Cell-mediated deposition of porous silica on bacterial biofilms. Biotechnol. Bioeng..

[226] Ferrer M.L., Yuste L., Rojo F., Del Monte F. (2003). Biocompatible sol-gel route for encapsulation of living bacteria in organically modified silica matrixes. Chem. Mater..

[227] Perullini M., Ferro Y., Durrieu C., Jobbagy M., Bilmes S.A. (2014). Sol gel silica platforms for microalgae-based optical biosensors. J. Biotechnol..

[228] McNulty M.J., Hamada N., Delzio J., McKee L., Nandi S., Longo M.L., McDonald K.A. (2022). Functionalizing silica sol-gel with entrapped plant virus-based immunosorbent nanoparticles. J. Nanobiotechnol..

[229] Wei Y., Xu J., Feng Q., Lin M., Dong H., Zhang W.J., Wang C. (2001). A novel method for enzyme immobilization: Direct encapsulation of acid phosphatase in nanoporous silica host materials. J. Nanosci. Nanotechnol..

[230] Singh S., Singhal R., Malhotra B.D. (2007). Immobilization of cholesterol esterase and cholesterol oxidase onto sol-gel films for application to cholesterol biosensor. Anal. Chim. Acta.

[231] Wallington S.A., Pilon C., Wright J.D. (1997). Sol-gel composites for optical sensing of solvents. J. Sol-Gel Sci. Technol..

[232] Crosley M.S., Yip W.T. (2017). Silica sol-gel optical biosensors: Ultrahigh enzyme loading capacity on thin films via kinetic doping. J. Phys. Chem. B.

[233] Wallace J.M., Dening B.M., Eden K.B., Stroud R.M., Long J.W., Rolison D.R. (2004). Silver-colloid-nucleated Cytochrome *c* superstructures encapsulated in silica nanoarchitectures. Langmuir.

[234] Wallace J.M., Stroud R.M., Pietron J.J., Long J.W., Rolison D.R. (2004). The effect of particle size and protein content on nanoparticle-gold-nucleated cytochrome c superstructures encapsulated in silica nanoarchitectures. J. Non-Cryst. Solids.

[235] Liu X., Zhang F., Jing X., Pan M., Liu P., Li W., Zhu B., Li J., Chen H., Wang L. (2018). Complex silica composite nanomaterials templated with DNA origami. Nature.

[236] Tan X.C., Tian Y.X., Cai P.X., Zou X.Y. (2005). Glucose biosensor based on glucose oxidase immobilized in sol-gel chitosan/silica hybrid composite film on Prussian blue modified glass carbon electrode. Anal. Bioanal. Chem..

[237] Liu S., Sun Y. (2007). Co-immobilization of glucose oxidase and hexokinase on silicate hybrid sol-gel membrane for glucose and ATP detections. Biosens. Bioelectron..

[238] Lloyd C.R., Eyring E.M. (2000). Protecting heme enzyme peroxidase activity from H_2_O_2_ inactivation by sol-gel encapsulation. Langmuir.

[239] Morosanova M.A., Morosanova E.I. (2023). Sol-gel films doped with enzymes and banana crude extract as sensing materials for spectrophotometric determination. Gels.

[240] Torres-Herrero B., Armenia I., Alleva M., Asin L., Correa S., Ortiz C., Fernandez-Alonso Y., Gutierrez L., de la Fuente J.M., Betancor L. (2023). Remote activation of enzyme nanohybrids for cancer prodrug therapy controlled by magnetic heating. ACS Nano.

[241] Keeling-Tucker T., Rakic M., Spong C., Brennan J.D. (2000). Controlling the material properties and biological activity of lipase within sol-gel derived bioglasses via organosilane and polymer doping. Chem. Mater..

[242] Eggers D.K., Valentine J.S. (2001). Molecular confinement influences protein structure and enhances thermal protein stability. Protein Sci..

[243] Luckarift H.R., Dickerson M.B., Sandhage K.H., Spain J.C. (2006). Rapid, room-temperature synthesis of antibacterial bionanocomposites of lysozyme with amorphous silica or titania. Small.

[244] Zheng L.L., Flora K., Brennan J.D. (1998). Improving the performance of a sol-gel-entrapped metal-binding protein by maximizing protein thermal stability before entrapment. Chem. Mater..

[245] Fennouh S., Guyon S., Livage J., Roux C. (2000). Sol-gel entrapment of *Escherichia coli*. J. Sol-Gel Sci. Technol..

[246] Mitchell R.J., Gu M.B. (2006). Characterization and optimization of two methods in the immobilization of 12 bioluminescent strains. Biosens. Bioelectron..

[247] Liu D.M., Chen I.W. (1999). Encapsulation of protein molecules in transparent porous silica matrices via an aqueous colloidal sol-gel process. Acta Mater..

[248] Shafiei N., Nasrollahzadeh M., Iravani S. (2021). Green synthesis of silica and silicon nanoparticles and their biomedical and catalytic applications. Comments Inorg. Chem..

[249] Nassif N., Coiffier A., Coradin T., Roux C., Livage J., Bouvet O. (2003). Viability of bacteria in hybrid aqueous silica gels. J. Sol-Gel Sci. Technol..

[250] Perullini M., Jobbagy M., Soler-Illia G.J.A.A., Bilmes S.A. (2005). Cell growth at cavities created inside silica monoliths synthesized by sol-gel. Chem. Mater..

[251] Perullini M., Rivero M.M., Jobbagy M., Mentaberry A., Bilmes S.A. (2007). Plant cell proliferation inside an inorganic host. J. Biotechnol..

[252] Jiang Y., Jiang Y.J., Mang Y.F., Li R., Zhang L., Jiang Z.Y. (2007). Biosilica-coated kappa-carrageenan microspheres for yeast alcohol dehydrogenase encapsulation. J. Biomater. Sci. Polymer Edn..

[253] Patnode W.I. (1942). Method of Bendering Materials Water Repellent. U.S. Patent.

[254] Cappelletti E.M., Carturan G., Piovan A. (1999). Production of Secondary Metabolites with Plant Cells Immobilized in a Porous Inorganic Support. U.S. Patent.

[255] Campostrini R., Carturan G., Caniato R., Piovan A., Filippin R., Innocenti G., Cappelletti E.M. (1996). Immobilization of plant cells in hybrid sol-gel materials. J. Sol-Gel Sci. Technol..

[256] Muraca M., Vilei M.T., Zanusso G.E., Ferraresso C., Boninsegna S., Dal Monte R., Carraro P., Carturan G. (2002). SiO_2_ entrapment of animal cells: Liver-specific metabolic activities in silica-overlaid hepatocytes. Artif. Organs..

[257] Eggers D.K., Valentine J.S. (2001). Crowding and hydration effects on protein conformation: A study with sol-gel encapsulated proteins. J. Mol. Biol..

[258] Brennan J.D., Benjamin D., DiBattista E., Gulcev M.D. (2003). Using sugar and amino acid additives to stabilize enzymes within sol-gel derived silica. Chem. Mater..

[259] Knorr L., Weyland H. (1916). Esters of Silicic Acid. U.S. Patent.

[260] Noll W. (1968). Chemistry and Technology of Silicones.

[261] Gill I., Ballesteros A. (1998). Encapsulation of biologicals within silicate, siloxane, and hybrid sol-gel polymers: An efficient and generic approach. J. Am. Chem. Soc..

[262] Khonina T.G., Chupakhin O.N., Larionov L.P., Boyakovskaya T.G., Suvorov A.L., Shadrina E.V. (2009). Synthesis, toxicity, and percutaneous activity of silicon glycerolates and related hydrogels. Pharm. Chem. J..

[263] Khonina T.G., Safronov A.P., Shadrina E.V., Ivanenko M.V., Suvorova A.I., Chupakhin O.N. (2012). Mechanism of structural networking in hydrogels based on silicon and titanium glycerolates. J. Colloid Interface Sci..

[264] Harper J.C., Lopez D.M., Larkin E.C., Economides M.K., McIntyre S.K., Alam T.M., Tartis M.S., Werner-Washburne M., Brinker C.J., Brozik S.M. (2011). Encapsulation of *S. cerevisiae* in poly(glycerol) silicate derived matrices: Effect of matrix additives and cell metabolic phase on long-term viability and rate of gene expression. Chem. Mater..

[265] Ishikawa Y., Sakamoto K., Toshihiko K., Yajima I., Takahashi S., Watanabe K. (2008). Water-Soluble Metal Alcoholate Derivative. Process for Production of the Derivative, and Solid Gelatinous Agent for External Applivation Comprising the Derivative. European Patent.

[266] Brook M.A., Chen Y., Guo K., Zhang Z., Jin W., Deisingh A., Cruz-Aguado J., Brennan J.D. (2004). Proteins entrapped in silica monoliths prepared from glyceroxysilanes. J. Sol-Gel Sci. Technol..

[267] Besanger T.R., Easwaramoorthy B., Brennan J.D. (2004). Entrapment of highly active membrane-bound receptors in macroporous sol-gel derived silica. Anal. Chem..

[268] Hodgson R.J., Chen Y., Zhang Z., Tleugabulova D., Long H., Zhao X.M., Organ M., Brook M.A., Brennan J.D. (2004). Protein-doped monolithic silica columns for capillary liquid chromatography prepared by the sol-gel method: Applications to frontal affinity chromatography. Anal. Chem..

[269] Hodgson R.J., Brook M.A., Brennan J.D. (2005). Capillary-scale monolithic immunoaffinity columns for immunoextraction with in-line laser-induced fluorescence detection. Anal. Chem..

[270] Rupcich N., Nutiu R., Li Y.F., Brennan J.D. (2005). Entrapment of fluorescent signaling DNA aptamers in sol-gel-derived silica. Anal. Chem..

[271] Besanger T.R., Hodgson R.J., Guillon D., Brennan J.D. (2006). Monolithic membrane-receptor columns: Optimization of column performance for frontal affinity chromatography/mass spectrometry applications. Anal. Chim. Acta.

[272] Cruz-Aguado J.A., Chen Y., Zhang Z., Brook M.A., Brennan J.D. (2004). Entrapment of Src protein tyrosine kinase in sugar-modified silica. Anal. Chem..

[273] Hui C.Y., Li Y., Brennan J.D. (2014). Fluorescence analysis of the properties of structure-switching DNA aptamers entrapped in sol-gel-derived silica materials. Chem. Mater..

[274] Carrasquilla C., Kapteyn E., Li Y., Brennan J.D. (2017). Sol-gel-derived biohybrid materials incorporating long-chain DNA aptamers. Angew. Chem. Int. Ed..

[275] Khonina T.G., Safronov A.P., Ivanenko M.V., Shadrina E.V., Chupakhin O.N. (2015). Features of silicon- and titanium-polyethylene glycol precursors in sol-gel synthesis of new hydrogels. J. Mater. Chem. B.

[276] Brandhuber D., Torma V., Raab C., Peterlik H., Kulak A., Husing N. (2005). Glycol-modified silanes in the synthesis of mesoscopically organized silica monoliths with hierarchical porosity. Chem. Mater..

[277] Tleugabulova D., Duft A.M., Zhang Z., Chen Y., Brook M.A., Brennan J.D. (2004). Evaluating formation and growth mechanisms of silica particles using fluorescence anisotropy decay analysis. Langmuir.

[278] Besanger T.R., Hodgson R.J., Green J.R.A., Brennan J.D. (2006). Immobilized enzyme reactor chromatography: Optimization of protein retention and enzyme activity in monolithic silica stationary phases. Anal. Chim. Acta.

[279] Goring G.L.G., Brennan J.D. (2007). Effect of ormosil and polymer doping on the morphology of separately and co-hydrolyzed silica films formed by a two-step aqueous processing method. Chem. Mater..

[280] Ivanenko M.V., Nikitina E.Y., Khonina T.G., Shadrina E.V., Novoselova M.E., Kuznetsov D.K., Karabanalov M.S. (2019). Features of formation and structure of silicon-polysaccharide-containing polyolate hydrogels obtained by the method of biomimetic mineralization. J. Sol-Gel Sci. Technol..

[281] Lavrova D.G., Zvonarev A.N., Alferov V.A., Khonina T.G., Shadrina E.V., Alferov S.V., Ponamoreva O.N. (2023). Biocompatible silica-polyethylene glycol-based composites for immobilization of microbial cells by sol-gel synthesis. Polymers.

[282] Cruz-Aguado J.A., Chen Y., Zhang Z., Elowe N.H., Brook M.A., Brennan J.D. (2004). Ultrasensitive ATP detection using firefly luciferase entrapped in sugar-modified sol-gel-derived silica. J. Am. Chem. Soc..

[283] Krieble R.H., Burkhard C.A. (1947). Cyclic dimethylpolymethylenedioxysilanes. J. Am. Chem. Soc..

[284] Sprung M.M. (1958). Reactions of di- and trifunctional methylsilanes with diols, triols, and fcyloxyols. J. Org. Chem..

[285] Mehrotra R.C., Narain R.P. (1967). Reactions of tetramethoxy- and triethoxysilanes with glycols. Indian J. Chem..

[286] Mehrotra R.C. (1966). Synthesis and properties of alkoxy- and acyloxysilanes. Pure Appl. Chem..

[287] Frye C.L. (1969). Stable silicon heterocyclic derivatives of branched alkane-diols. J. Org. Chem..

[288] Frye C.L. (1970). Pentacoordinate silicon derivatives. IV. Alkylammonium siliconate salts derived from aliphatic 1,2-diols. J. Am. Chem. Soc..

[289] Mehrotra R.C., Pant B.C. (1964). Organic derivatives of silicon.Part V.Reactions of silicon tetra-acetate with glycols:Synthesis of glycol derivatives of silicon. J. Indian Chem. Soc..

[290] Kuznetsova V.P., Belogolovina G.N. (1969). Synthesis of hydrohyalkoxysilames and urethans derived from them. J. Gen. Chem. USSR Eng. Transl..

[291] Sattler K., Gradzielski M., Mortensen K., Hoffmann H. (1998). Influence of surfactant on the gelation of novel ethylene glycol esters of silicic acid. Ber. Bunsenges. Phys. Chem..

[292] Sattler K., Hoffmann H. (1999). A novel glycol silicate and its interaction with surfactant for the synthesis of mesoporous silicate. Prog. Colloid Polym. Sci..

[293] Husing N., Brandhuber D., Kaiser P. (2006). Glycol-modified organosilanes in the synthesis of inorganic-organic silsesquioxane and silica monoliths. J. Sol-Gel Sci. Technol..

[294] Hartmann S., Brandhuber D., Husing N. (2007). Glycol-modified silanes: Novel possibilities for the synthesis of hierarchically organized (hybrid) porous materials. Acc. Chem. Res..

[295] Hartmann S. (2009). Hierarchically Organized (Hybrid) Silica Monoliths for the Application as Stationary Phases in HPLC. Ph.D. Thesis.

[296] Bravo-Flores I., Melendez-Zamudio M., Guerra-Contreras A., Ramirez-Oliva E., Alvarez-Guzman G., Zarraga-Nunez R., Villegas A., Cervantes J. (2021). Revisiting the system silanes-polysaccharides: The cases of THEOS-chitosan and MeTHEOS-chitosan. Macromol. Rapid Commun..

[297] Cheng H., Tamaki R., Laine R.M., Babonneau F., Chujo Y., Treadwell D.R. (2000). Neutral alkoxysilanes from silica. J. Am. Chem. Soc..

[298] Jitchum V., Chivin S., Wongkasemjit S., Ishida H. (2001). Synthesis of spirosilicates directly from silica and ethylene glycol/ethylene glycol derivatives. Tetrahedron.

[299] Meyer M., Fischer A., Hoffmann H. (2002). Novel ringing silica gel that do not shrink. J. Phys. Chem. B.

[300] Husing N., Raab C., Torma V., Roig A., Peterlik H. (2003). Periodically mesostructured silica monoliths from diol-modified silanes. Chem. Mater..

[301] Thitinun S., Thanabodeekij N., Jamieson A., Wongkasemjit S. (2003). Sol-gel processing of spirosilicates. J. Eur. Ceram. Soc..

[302] Shchipunov Y.A. (2003). Sol-gel derived biomaterials of silica and carrageenans. J. Colloid Interface Sci..

[303] Shchipunov Y.A., Karpenko T.Y. (2004). Hybrid polysaccharide-silica nanocomposites prepared by the sol-gel technique. Langmuir.

[304] Shchipunov Y.A., Kojima A., Imae T. (2005). Polysaccharides as a template for silicate generated by sol-gel processes. J. Colloid Interface Sci..

[305] Bendedouch D., Chen S.H. (1983). Structure and interparticle interactions of bovine serum albumin in solution studied by small-angle neutron scattering. J. Phys. Chem..

[306] Peters T. (1985). Serum albumin. Adv. Protein Chem..

[307] Flory P.J. (1953). Principles of Polymer Chemistry.

[308] Tirrell M., Goddard E.D., Ananthapadmanabhan K.P. (1993). Fundamentals of polymer solutions. Interactions of Surfactants with Polymers and Proteins.

[309] Jonsson B., Lindman B., Holmberg K., Kronberg B. (2002). Surfactants and Polymers in Aqueous Solutions.

[310] Morris E.R., Norton I.T. (1983). Polysaccharide aggregation in solutions and gels. Stud. Phys. Theor. Chem..

[311] Peppas N.A., Sahlin J.J. (1996). Hydrogels as mucoadhesive and bioadhesive materials: A review. Biomaterials.

[312] Lee K.Y., Mooney D.J. (2001). Hydrogels for tissue engineering. Chem. Rev..

[313] Mano J.F., Silva G.A., Azevedo H.S., Malafaya P.B., Sousa R.A., Silva S.S., Boesel L.F., Oliveira J.M., Santos T.C., Marques A.P. (2007). Natural origin biodegradable systems in tissue engineering and regenerative medicine: Present status and some moving trends. J. R. Soc. Interface.

[314] Schnepp Z. (2013). Biopolymers as a flexible resource for nanochemistry. Angew. Chem. Int. Ed..

[315] Lim K.S., Martens P., Poole-Warren L., Li J., Osada Y., Cooper-White J. (2018). Biosynthetic hydrogels for cell encapsulation. Functional Hydrogels as Biomaterials.

[316] Tardy B.L., Mattos B.D., Otoni C.G., Beaumont M., Majoinen J., Kamarainen T., Rojas O.J. (2021). Deconstruction and reassembly of renewable polymers and biocolloids into next generation structured materials. Chem. Rev..

[317] Lin X., Wang J.L., Wu X.Y., Luo Y., Wang Y.A., Zhao Y.J. (2023). Marine-derived hydrogels for biomedical applications. Adv. Funct. Mater..

[318] Schante C.E., Zuber G., Herlin C., Vandamme T.F. (2011). Chemical modifications of hyaluronic acid for the synthesis of derivatives for a broad range of biomedical applications. Carbohyd. Polym..

[319] Jayakumar R., Prabaharan M., Sudheesh Kumar P.T., Nair S.V., Tamura H. (2011). Biomaterials based on chitin and chitosan in wound dressing applications. Biotechnol. Adv..

[320] Fourmentin S., Crini G., Lichtfouse E. (2018). Cyclodextrin Fundamentals, Reactivity and Analysis.

[321] Takeshita S., Zhao S., Malfait W.J., Koebel M.M. (2021). Chemistry of chitosan aerogels: Three-dimensional pore control for tailored applications. Angew. Chem. Int. Ed..

[322] Shchipunov Y.A., Karpenko T.Y., Krekoten A.V., Postnova I.V. (2005). Gelling of otherwise nongelable polysaccharides. J. Colloid Interface Sci..

[323] Gruber J.V., Goddard E.D., Gruber J.V. (1999). Polysaccharide-based polymers in cosmetics. Principle of Polymer Science and Technology in Cosmetics and Personal Care.

[324] Chambon F., Winter H.H. (1987). Linear viscoelasticity at the gel point of a crosslinking PDMS with imbalanced stoichiometry. J. Rheol..

[325] Shchipunov Y.A., Krekoten A.V., Kuryavyi V.G., Topchieva I.N. (2005). Microporous nanocomposite material synthesized by sol-gel processing in the presence of cyclodextrins. Colloid J..

[326] Szejtli J. (1998). Introduction and general overview of cyclodextrin chemistry. Chem. Rev..

[327] Han B.H., Polarz S., Antonietti M. (2001). Cyclodextrin-based porous silica materials as in situ chemical “nanoreactors” for the preparation of variable metal-silica hybrids. Chem. Mater..

[328] Han B.H., Antonietti M. (2002). Cyclodextrin-based pseudopolyrotaxanes as templates for the generation of porous silica materials. Chem. Mater..

[329] Han B.H., Antonietti M. (2003). One-step synthesis of copper nanoparticles containing mesoporous silica by nanocasting of binuclear copper(II) complexes with cyclodextrins. J. Mater. Chem..

[330] Wang G.H., Zhang L.M. (2007). Manipulating formation and drug-release behavior of new sol-gel silica matrix by hydroxypropyl guar gum. J. Phys. Chem. B.

[331] Korpi A., Kostiainen M.A. (2022). Sol-gel synthesis of mesoporous silica using a protein crystal template. ChemNanoMat.

[332] Clark A.H., Hill S.E., Ledward D.A., Mitchell J.R. (1998). Gelation of globular proteins. Functional Properties of Food Macromolecules.

[333] Wang G.H., Zhang L.M. (2009). A biofriendly silica gel for in situ protein entrapment: Biopolymer-assisted formation and its kinetic mechanism. J. Phys. Chem. C.

[334] Shchipunov Y.A., Shipunova N.Y. (2008). Regulation of silica morphology by proteins serving as a template for mineralization. Colloid Surf. B.

[335] Shkryl Y.N., Bulgakov V.P., Veremeichik G.N., Kovalchuk S.N., Kozhemyako V.B., Kamenev D.G., Semiletova I.V., Timofeeva Y.O., Shchipunov Y.A., Kulchin Y.N. (2016). Bioinspired enzymatic synthesis of silica nanocrystals provided by recombinant silicatein from the marine sponge *Latrunculia oparinae*. Bioproc. Biosyst. Eng..

[336] Guenet J.M. (1992). Thermoreversible Gelation of Polymers and Biopolymers.

[337] De Wolf F.A. (2003). Collagen and gelatin. Prog. Biotechol..

[338] Shkryl Y.N., Semiletova I.V., Nepomnyaschiy A.V., Kovalchuk S.N., Veremeichik G.N., Avramenko T.V., Bulgakov V.P., Shchipunov Y.A., Voznesensky S.S., Kozhemyako V.B. (2018). Biomimetic synthesis of nanosized silica structures on a substrate with silicatein. Russ. J. Bioorgan. Chem..

[339] Mitra A., Imae T., Shchipunov Y.A. (2005). Fibrous silica composite fabricated by the sol-gel processing on aggregates of amino acid surfactant. J. Sol-Gel Sci. Technol..

[340] Takahashi S., Ikkai Y., Rodriguez-Abreu C., Aramaki K., Ohsuna T., Sakamoto K. (2007). Application of a water soluble alkoxysilane for the formation of mesoporous silica from nonionic surfactant micelles bearing cholesterol. Chem. Lett..

[341] Shchipunov Y.A., Karpenko T.Y., Bakunina I.Y., Burtseva Y., Zvyagintseva T.N. (2004). A new precursor for the Immobilization of enzymes inside sol-gel derived hybrid silica nanocomposites containing polysaccharides. J. Biochem. Biophys. Methods.

[342] Shchipunov Y.A., Burtseva Y.V., Karpenko T.Y., Shevchenko N.M., Zvyagintseva T.N. (2006). Highly efficient immobilization of endo-1,3--d-glucanases (laminarinases) from marine mollusks in novel hybrid polysaccharidesilica nanocomposites with regulated composition. J. Mol. Catal. B Enzym..

[343] Bakunina I.Y., Nedashkovskaya O.I., Zvyagintseva T.N., Shchipunov Y.A. (2006). Immobilization of alpha-galactosidase inside hybrid silica nanocomposites containing polysaccharides. Russ. J. Appl. Chem..

[344] Solov’eva T.F., Elyakova L.A., Zvyagintseva T.N., Yermak I.M. (1996). Polysaccharides from the Russia Pacific coast algae and their enzymatic transformation. J. Marine Technol. Soc..

[345] Zvyagintseva T.N., Elyakova L.A., Isakov V.V. (1995). The enzymatic transformations of laminarans in 1,3; 1,6--d-glucans with immunostimulating activity. Bioorg. Khim..

[346] Wang G.H., Zhang L.M. (2006). Using novel polysaccharide-silica hybrid material to construct an amperometric biosensor for hydrogen peroxide. J. Phys. Chem. B.

[347] Zhang L.M., Wang G.H., Xing Z. (2011). Polysaccharide-assisted incorporation of multiwalled carbon nanotubes into sol-gel silica matrix for electrochemical sensing. J. Mater. Chem..

[348] Wang G.H., Zhang L.M. (2018). Electroactive polyaniline/silica hybrid gels: Controllable sol-gel transition adjusted by chitosan derivatives. Carbohyd. Polym..

[349] Shchipunov Y.A., Chesnokov A.V. (2003). Carrageenan gels in skim milk: Formation and rheological properties. Colloid J..

[350] Eltzov E., Marks R.S. (2010). Fiber-optic based cell sensors. Adv. Biochem. Engin. Biotechnol..

[351] Voznesensky S.S., Popik A.Y., Gamayunov E.L., Orlova T.Y., Markina Z., Postnova I.V., Shchipunov Y. (2018). One-stage immobilization of the microalga *Porphyridium purpureum* using a biocompatible silica precursor and study of the fluorescence of its pigments. Eur. Biophys. J..

[352] Moreno-Garrido I. (2008). Microalgae immobilization: Current techniques and uses. Bioresour. Technol..

[353] Nguyen-Ngoc H., Tran-Minh C. (2007). Fluorescent biosensor using whole cells in an inorganic translucent matrix. Anal. Chim. Acta.

[354] Lee R.E. (2008). Phycology.

[355] Brayner R., Coute A., Livage J., Perrette C., Sicard C. (2011). Micro-algal biosensors. Anal. Bioanal. Chem..

[356] Postnova I.V., Sarin S.A., Karpenko T.Y., Shchipunov Y.A. (2020). Formation of photocatalytically active titania on mesoporous silica with silver nanoparticles synthesized using tannin as a template and a reductant. Dokl. Chem..

[357] Postnova I., Shchipunov Y. (2022). Tannic acid as a versatile template for silica monoliths engineering with catalytic gold and silver nanoparticles. Nanomater.

[358] Dos Santos C., Vargas A., Fronza N., dos Santos J.H.Z. (2017). Structural, textural and morphological characteristics of tannins from Acacia mearnsii encapsulated using sol-gel methods: Applications as antimicrobial agents. Colloid Surf. B.

[359] Pelton M., Aizpurua J., Bryant G. (2008). Metal-nanoparticle plasmonics. Laser Photonics Rev..

[360] Mayer K.M., Hafner J.H. (2011). Localized surface plasmon resonance sensors. Chem. Rev..

[361] Quiquempois Y., Niay P., Douay M., Poumellec B. (2003). Advances in poling and permanently induced phenomena in silica-based glasses. Curr. Opin. Solid State Mater. Sci..

[362] Pruneri V., Bonfrate G., Kazansky P.G., Takebe H., Morinaga K., Kohno M., Kuwasaki K., Takeuchi T. (1999). High second-order optical nonlinearities in thermally poled sol-gel silica. Appl. Phys. Lett..

[363] Kulchin Y.N., Bezverbny A.V., Bukin O.A., Voznesensky S.S., Golik S.S., Mayor A.Y., Shchipunov Y.A., Nagorny I.G. (2011). Nonlinear optical properties of biomineral and biomimetical nanocomposite structures. Laser Phys..

[364] Postnova I., Bezverbny A., Golik S., Kulchin Y., Li H., Wang J., Kim I., Ha C.S., Shchipunov Y. (2012). Tailored hybrid hyperbranched polyglycidol-silica nanocomposites with high third-order nonlinearity. Int. Nano Lett..

[365] Li H., Jo J.K., Zhang L.D., Ha C.S., Suh H., Kim I. (2010). Hyperbranched polyglycidol assisted green synthetic protocols for the preparation of multifunctional metal nanoparticles. Langmuir.

[366] Kulchin Y., Golik S., Proschenko D., Chekhlenok A., Postnova I.V., Mayor A., Shchipunov Y. (2013). Supercontinuum generation and filamentation of ultrashort laser pulses in hybrid silicate nanocomposite materials on the basis of polysaccharides and hyperbranched polyglycidols. Quantum Electron..

[367] Kulchin Y., Mayor A., Proschenko D., Chekhlenok A., Postnova I.V., Golik S., Bukin O., Shchipunov Y. (2014). Threshold energies for filamentation and spectral characteristics of supercontinuum generation in THEOS-based nanocomposite organosilicon media. Quantum Electron..

[368] Proschenko D., Mayor A., Bukin O., Golik S., Chekhlenok A., Postnova I., Shchipunov Y.A., Kulchin Y. (2014). Interaction of the femtosecond laser pulses with the new silica nanocomposites containing Au and CdS. Adv. Mater. Res..

[369] Kulchin Y.N., Mayor A.Y., Proschenko D.Y., Shchipunov Y.A. (2016). Nonlinear optical properties and supercontinuum spectrum of titania-modified carbon quantum dots. Quantum Electron..

[370] Sergeeva K.M., Postnova I.V., Shchipunov Y.A. (2013). Entrapment of quantum dots in silica matrix with compatible precursor. Colloid J..

[371] Voznesensky S.S., Sergeev A.A., Galkina A.N., Kulchin Y., Shchipunov Y., Postnova I.V. (2014). Laser-induced photodynamic effects at silica nanocomposite based on cadmium sulphide quantum dots. Opt. Express.

[372] Voznesenskiy S., Sergeev A., Postnova I.V., Galkina A., Shchipunov Y., Kulchin Y. (2015). Dynamic laser-induced effects in nanocomposite systems based on the cadmium sulfide quantum dots in a silicate matrix. Opt. Express.

[373] Postnova I., Voznesenskiy S., Sergeev A., Galkina A., Kulchin Y., Shchipunov Y. (2018). Photonic materials prepared through the entrapment of quantum dots into silica. Colloid Surf. A.

[374] Shchipunov Y.A., Krekoten A.V., Petukhova M.V. (2008). Luminescent nanocomposite synthesized by sol-gel method in micellar solution of alkylpolyglycoside with solubilized luminol. Colloid J..

[375] Shchipunov Y.A., Khlebnikov O.N. (2011). Nanocomposite material with immobilized acid-base dyes conjugated with polysaccharides. Colloid J..

[376] Qin Y., Wang J., Qiu C., Hu Y., Xu X., Jin Z. (2019). Self-assembly of metal-phenolic networks as functional coatings for preparation of antioxidant, antimicrobial, and pH-sensitive-modified starch nanoparticles. ACS Sustain. Chem. Eng..

[377] Ali A., Hussain F., Attacha S., Kalsoom A., Qureshi W.A., Shakeel M., Militky J., Tomkova B., Kremenakova D. (2021). Development of novel antimicrobial and antiviral green synthesized silver nanocomposites for the visual detection of Fe^3+^ Ions. Nanomater.

[378] Said S., Wang T., Feng Y.N., Ren Y., Zhao Z.P. (2022). Recent progress in membrane technologies based on metal-phenolic networks: A review. Ind. Eng. Chem. Res..

